# 2D Re‐Based Transition Metal Chalcogenides: Progress, Challenges, and Opportunities

**DOI:** 10.1002/advs.202002320

**Published:** 2020-10-25

**Authors:** Xiaobo Li, Chao Chen, Yang Yang, Zhibin Lei, Hua Xu

**Affiliations:** ^1^ Key Laboratory of Applied Surface and Colloid Chemistry Ministry of Education Shaanxi Key Laboratory for Advanced Energy Devices School of Materials Science and Engineering Shaanxi Normal University Xi'an 710119 P. R. China

**Keywords:** 2D materials, anisotropy, optoelectronics, ReS_2_, transition metal chalcogenides

## Abstract

The rise of 2D transition‐metal dichalcogenides (TMDs) materials has enormous implications for the scientific community and beyond. Among TMDs, ReX_2_ (X = S, Se) has attracted significant interest regarding its unusual 1T′ structure and extraordinary properties in various fields during the past 7 years. For instance, ReX_2_ possesses large bandgaps (ReSe_2_: 1.3 eV, ReS_2_: 1.6 eV), distinctive interlayer decoupling, and strong anisotropic properties, which endow more degree of freedom for constructing novel optoelectronic, logic circuit, and sensor devices. Moreover, facile ion intercalation, abundant active sites, together with stable 1T′ structure enable them great perspective to fabricate high‐performance catalysts and advanced energy storage devices. In this review, the structural features, fundamental physicochemical properties, as well as all existing applications of Re‐based TMDs materials are comprehensively introduced. Especially, the emerging synthesis strategies are critically analyzed and pay particular attention is paid to its growth mechanism with probing the assembly process of domain architectures. Finally, current challenges and future opportunities regarding the controlled preparation methods, property, and application exploration of Re‐based TMDs are discussed.

## Graphical abstract

A review of 2D Re‐based transition metal chalcogenides (TMDs) is presented to introduce their crystal and electronic band structures, and to highlight the recent progress on fabrications, properties, and applications. In addition, the current challenges and future opportunities of ReX_2_ are discussed. This review provides a comprehensive summary about Re‐based TMDs, and sheds light on the current direction of research in this field.

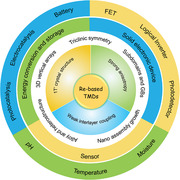

## 1 Introduction

Over the past decade, the superlative properties of graphene have inspired explosive interest in exploration of other 2D materials, initially focusing on transition metal dichalcogenides (TMDs) and recently broadening to a larger family of layered and nonlayered materials.^[^ ^1^, ^2^, ^3^ ^]^ 2D TMDs are MX_2_‐type compounds where M is a transition element from the periodic table and X represents the chalcogen species S, Se, and Te. The abundant transition elements from group‐IV to group‐X make the TMDs compounds extensive, which span the wide range of electronic structures, from semiconductor to metal/semimetal and even superconductor.^[^ ^4^, ^5^, ^6^ ^]^ Among these materials, group‐VI TMDs, such as MoS_2_ and WSe_2_, are of special interest since their diverse and controlled optical and electronic properties. Most notably, the indirect to direct transition of MoS_2_ from bulk to monolayer offers the feasibility to modulate its properties by tuning the thickness.^[^ ^7^, ^8^ ^]^ Besides, the broken spatial inversion symmetry and strong spin‐orbit coupling afford novel valley pseudospin properties in TMDs, which is crucial in exploring new spintronic and valleytronic materials and devices.^[^ ^9^ ^]^ The excellent mechanical strength, strong interaction with light, superior electrical properties, combined with naturally layered structures and large specific surface area, provide tremendous opportunities for applying 2D TMDs in next‐generation quantum information and communication devices, as well as energy conversion and storage devices, etc.^[^ ^10^, ^11^, ^12^ ^]^

Aside from the most popular group‐VI TMDs, group‐VII TMDs such as ReX_2_ (X = S, Se) has recently attracted significant interest for its wholly unusual features in all aspects of structural, electrical, optical, and chemical properties.^[^ ^13^, ^14^ ^]^ Unlike group‐VI TMDs that stabilized in highly symmetric 2H structure, ReX_2_ crystallizing in the unique distorted 1T (1T′) structure with reduced in‐plane crystal symmetry, which endow them strong in‐plane anisotropic optical, electrical, and phonon properties.^[^ ^15^, ^16^, ^17^, ^18^ ^]^ These extraordinary features are especially attractive for constructing novel photonic, electronic, and optoelectronic devices beyond conventional isotropic 2D materials.^[^ ^19^, ^20^, ^21^, ^22^, ^23^, ^24^ ^]^ Moreover, the weak interlayer coupling feature enables bulk ReX_2_ to behave as electronically and vibrationally decoupled monolayers, leading to an obvious layer‐independent character in various properties.^[^ ^13^, ^25^ ^]^ This distinctive character has brought many emerging applications, including catalytic reactions, rechargeable batteries, stimuli‐responsive devices, etc.^[^ ^26^, ^27^, ^28^ ^]^ Furthermore, the low‐symmetry structure of ReX_2_ brings both opportunities and challenges for the integration of it with the aforementioned high‐symmetry 2D materials to build novel alloys and heterostructures.^[^ ^29^, ^30^ ^]^ Besides ReX_2_, there are also several other attracting anisotropic 2D materials like phosphorene, GeAs and GeP materials are widely studied.^[^ ^31^, ^32^, ^33^ ^]^ Despite these materials possess broader bandgap variability and high‐performance electrical properties, ReX_2_ exhibit several advantages compared with these materials. For example, ReX_2_ has excellent environmental stability in ambient air, especially, it can be controlled prepared in large scale. Given the unusual structural features, better structural stability, outstanding properties, promising applications, and controllable preparation, ReX_2_ has now become one of the most attractive research topics in the 2D world.

Although there are several early reviews have introduced the research progress of ReS_2_,^[^ ^34^, ^35^, ^36^, ^37^ ^]^ the complete and systematic studies of novel properties and applications about ReX_2_ have been explored just in recent years. Moreover, the requirements for different functions of ReX_2_ stimulate the booming development of various synthetic techniques, and an entirely new cognition to the crystal growth behavior has been studying. Especially, in the wake of the deepening of ReX_2_ research, many new challenges and opportunities ahead for researchers, which will further motivate us to devote persistent efforts. Hence, a timely comprehensive review is of special significance to afford the latest advances and perspectives about this attractive material.

**Figure 1:**
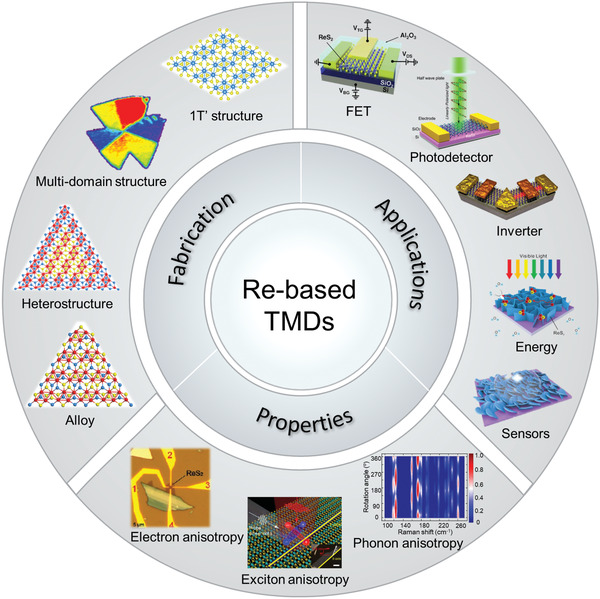
Overview of Re‐based TMDs showing the fabrications, properties, and relevant applications. (Images) Reproduced with permission.^[^ ^26^ ^]^ Copyright 2018, Wiley‐VCH; Reproduced with permission.^[^ ^19^ ^]^ Copyright 2015, Springer Nature; Reproduced with permission.^[^ ^28^ ^]^ Copyright 2018, Wiley‐VCH; Reproduced with permission.^[^ ^14^ ^]^ Copyright 2016, Springer Nature; Reproduced with permission.^[^ ^15^ ^]^ Copyright 2015, American Chemical Society; Reproduced with permission.^[^ ^132^ ^]^ Copyright 2016, Wiley‐VCH; Reproduced with permission.^[^ ^152^ ^]^ Copyright 2018, Wiley‐VCH; Reproduced with permission.^[^ ^38^ ^]^ Copyright 2019, Wiley‐VCH.

Here, this review provides a systematic introduction to the research progress of 2D Re‐based TMDs materials as schematically shown in **Figure** **1**. Initially, we outline their unusual structure features, booming properties and various preparation approaches especially focus on their chemical vapor deposition (CVD) growth progresses. Additionally, based on the structural and functional features of the Re‐based TMDs, we categorize all reported applications into five main areas, including i) electronics, ii) optoelectronic, iii) energy conversion and storage, iv) surface‐enhanced Raman spectra, and v) sensors. Each subsection is organized from the brief introduction of basic concepts following by some typical applications. Finally, current challenges and future perspectives regarding the controlled preparation, property, and application exploration of ReX_2_ are discussed.

## 2 Structure and Properties of ReX_2_

### 2.1 Crystal Structure

**Figure 2:**
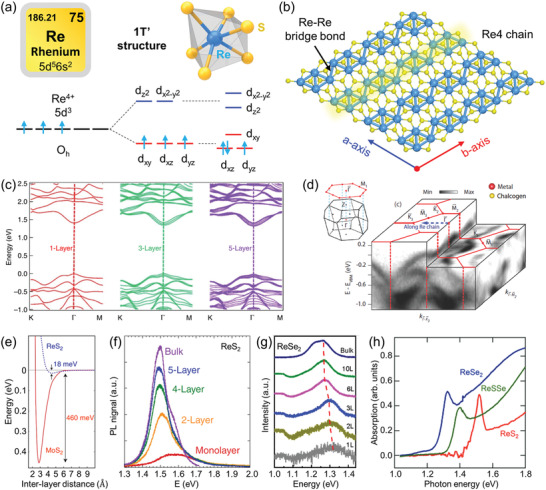
a) Valence electron structure of Re in 1T′ ReS_2_ crystal. b) Schematic atomic structure of monolayer ReX_2_. Reproduced with permission.^[^ ^38^ ^]^ Copyright 2019, Wiley‐VCH. c) DFT calculated electronic band structure of monolayer (orange curves), trilayer (orange curves), and five‐layer (orange solid curves) ReS_2_. Reproduced with permission.^[^ ^19^ ^]^ Copyright 2015, Springer Nature. d) Overview of the valence‐band structure as measured by ARPES. The surface Brillouinzone is shown as red lines, and the momentum space direction corresponding to the real‐space direction along the Re chains is also indicated. The bulk and projected surface Brillouin zones are shown in the inset. Reproduced with permission.^[^ ^47^ ^]^ Copyright 2017, American Physical Society. e) The calculated total energy of the system as a function of interlayer separation. The significantly shallower depth of the well in ReS_2_ implies much weaker interlayer coupling energy in ReS_2_ as compared with MoS_2_. f) PL spectrum of ReS_2_ with different number of layers. Reproduced with permission.^[^ ^13^ ^]^ Copyright 2014, Springer Nature. g) PL spectrum of ReSe_2_ with different number of layers. Reproduced with permission.^[^ ^56^ ^]^ Copyright 2015, Tsinghua University Press and Springer Nature. h) Absorption spectrum of ReS_2_, ReSSe alloy, and ReSe_2_. Reproduced with permission.^[^ ^58^ ^]^ Copyright 2016, Royal Society of Chemistry.

Rhenium, as one of the rarest elements in the earth's crust, belongs to the transition metal of the sixth period in the periodic table with the symbol Re and atomic number 75. **Figure** **2**a shows the valence electron structure of Re atom in ReX_2_ crystal and its schematic atomic structure.^[^ ^38^ ^]^ The Re atom in ReS_2_ has a valence of +4 with the extranuclear electron configuration of Re^4+^ (5d^3^) and forms six covalent bonds with the surrounding S atoms. Unlike the most popular group‐VI TMDs (such as MoS_2_ and WS_2_) which crystallize in the hexagonal (2H) phase, an extra valence electron of Re^4+^ occupies in the degenerate states of t_2g_ is energetically unfavorable. Due to the Jahn‐Teller effect (or Peierls distortion), the t_2g_ energy levels in the octahedral field will further split to break the degeneracy, leading ReS_2_ as a distorted octahedral (1T′) structure, in which the threefold rotational symmetry of the parent 1T structure is broken.^[^ ^39^, ^40^, ^41^ ^]^ As a result, the adjacent Re atoms are bonded in the form of zigzag Re4 clusters and aligned along the direction of the lattice vector *b* to form a Re4‐chain which is defined as the *b*‐axis of ReX_2_ crystal (Figure 2b). This distinguishing crystal structure endows ReX_2_ crystal with many novel properties for diverse applications. The lattice parameters and space groups of ReX_2_ (ReS_2_ and ReSe_2_) are summarized in **Table** **1**.^[^ ^42^ ^]^ In theory, the stability of layered structure materials is thermodynamically preferred with the d‐electron count of the transition metal.^[^ ^39^ ^]^ For d^0^, the octahedral structure is more stable, then filling the lowest d bands starts to favor the trigonal‐prismatic structure. For still larger d‐electron counts again the octahedral geometry is more stable and the crossover of the two effects is at the d‐electron equal to 2. When electron counts over d^3^, the stability becomes worse for the Fermi surface of the 1T phase is partially or weakly nested. Thus, for the ReX_2_ with d^3^ coordination, the undistorted 1T phase is not stable. In this regard, a periodic distortion of crystal lattice is adopted. The splitting of partially filled degenerate orbital causes a reduction of the free energy in 1T′ phase ReX_2_, resulting in it to form a stable 1T′ phase. Therefore, the most stable phase structure of ReX_2_ should be the distorted 1T phase (1T′ phase), and nearly all presently reported experimental works on ReX_2_ prepared by either CVT or CVD are 1T′ phase structure as discussed below. Certainly, recent theory works have proposed new phase (Tri phase) of ReS_2_, and they predicted that the transition from 1T′ to Tri phase can be induced with either electron doping or lithium atom intercalating.^[^ ^43^ ^]^ Moreover, the full‐surface hydrogenation can induce the structural transition of single layer ReS_2_ from 1T′ to 1T‐ReS_2_H_2_ which is predicted as an indirect‐gap semiconductor.^[^ ^44^ ^]^ However, the new phase structure of ReS_2_ has not been realized in experiment, which deserve to be further explored in the future. Moreover, the 1T′ ReS_2_ can stable in ambient air for about 2–3 years or even longer, showing much high environmental stability, which is different from the frail environment stability of phosphorus and 1T′ MoTe_2_/WTe_2_.^[^ ^45^ ^]^

**Table 1:** Unit‐cell dimensions of ReS_2_ and ReSe_2_. *Z* is the number of formula units per cell. *a* and *b* are the in‐plane axes. The space group is *P‐1* ^[^ ^42^ ^]^

	*a* [Å]	*b* [Å]	*c* [Å]	*α* [deg]	*β* [deg]	*γ* [deg]	*Z*
ReS_2_	6.352	6.446	12.779	91.51	105.17	118.97	8
ReSe_2_	6.597	6.710	6.721	91.84	104.90	118.91	4

### 2.2 Electronic Band Structure

The valence band and conduction band edges of ReX_2_ are contributed by the d‐orbitals of Re atoms and p‐orbitals of X atoms. Density functional theory (DFT) calculations show that ReS_2_ nanosheets with thickness from bulk to monolayer have almost the same band structures except a minor bandgap widening.^[^ ^13^ ^]^ Especially, all ReS_2_ with different thickness belong to the direct bandgap semiconductors with bandgap of 1.35 eV for bulk, 1.40 eV for trilayer, and 1.44 eV for monolayer (Figure 2c).^[^ ^19^ ^]^ This is a significant difference compared with other 2D TMDs where the electronic band structures show strongly layer number dependence and undergo a transition from indirect to direct bandgap as their thickness reduced from bulk to monolayer.^[^ ^8^ ^]^ This stark difference is attributed to the weaker interlayer coupling of ReS_2_ induced by the distorted 1T structure, which leads to the absence of band renormalization, and eventually allows bulk ReS_2_ behave as electronically and vibrationally decoupled monolayers.^[^ ^13^ ^]^ Notably, the electronic band structure of ReSe_2_ is obviously different from that of ReS_2_. Wolverson et al. found that the bulk ReSe_2_ behaves as a direct bandgap (1.09–1.31 eV) semiconductor via projector augmented wave calculation, while single layer ReSe_2_ has an indirect bandgap (1.34 eV).^[^ ^46^ ^]^ Angle‐resolved photoemission spectroscopy (ARPES) was utilized to provide an accurate description of the electronic band structure of ReS_2_, as shown in Figure 2d.^[^ ^47^ ^]^ The k_z_ dispersion of the band‐edge states explains the observed tiny widening of bandgap when ReS_2_ is thinned from bulk to monolayer, as quantum confinement in the z direction will increase the energy of the direct bandgap in ReS_2_. Moreover, though the surface Brillouin zone of ReS_2_ remains almost hexagonal, its electronic structure presents obvious anisotropic feature along different in‐plane directions, which brings many novel anisotropic optical and electrical properties as discussed in the following section.

Energy band engineering of ReX_2_, which is critical to fulfilling the requirements of diverse optoelectronics, has been widely explored via strain, doping, molecular modification or high pressure.^[^ ^48^, ^49^, ^50^, ^51^ ^]^ For instance, theory calculations and experiments have shown that both the compressive and tensile strain applied in different lattice directions of ReX_2_ can modulate the electronic band structures, and the bandgap is monotonically reduced with increasing strain in each direction. In addition, the high pressure can induce metallization and superconducting phase in ReS_2_ and ReSe_2_ with a band overlap mechanism.

### 2.3 Optical and Vibration Properties

DFT calculations demonstrate that the interlayer coupling energy of ReS_2_ is about 18 meV per unit cell, while that of MoS_2_ is about 460 meV for the 2 × 2 conventional cell (Figure 2e),^[^ ^13^ ^]^ indicating a much weak interlayer coupling of ReS_2_. Such weak interlayer coupling of ReS_2_ compared with other 2D materials (like MoS_2_, GeSe_2_, BP, SnS and PtSe_2_, etc.) are also confirmed via the thickness‐dependent Raman and fluorescence spectra, the temperature‐dependent Raman spectra, and the force constants per unit of low frequency modes.^[^ ^52^, ^53^, ^54^, ^55^ ^]^ For example, the PL spectra of ReX_2_ shows negligible layer number dependence. With the thickness of ReS_2_ and ReSe_2_ thining from bulk to monolayer, their PL intensity decreases along with negligible blueshift of peak position (Figure 2f,g),^[^ ^13^, ^56^ ^]^ which is consistent well with theoretical bandgap. This result indicates that thinning down the flake does not enhance the quantum confinement of electron, and the neighbouring monolayers in the flake are largely electronically decoupled. In the optical absorption spectra, three optical transitions were identified for both bulk and monolayer ReS_2_, indicating decoupled excitonic and emission properties.^[^ ^57^ ^]^ Moreover, the exciton peaks of ReS_2_ (1.51 eV) and ReSe_2_ (1.32 eV) are corresponding well with the bandgap achieved from their PL spectra, and that of ReSSe alloy located at 1.39 eV demonstrate the effective energy band engineering of their alloys (Figure 2h).^[^ ^58^ ^]^

**Figure 3:**
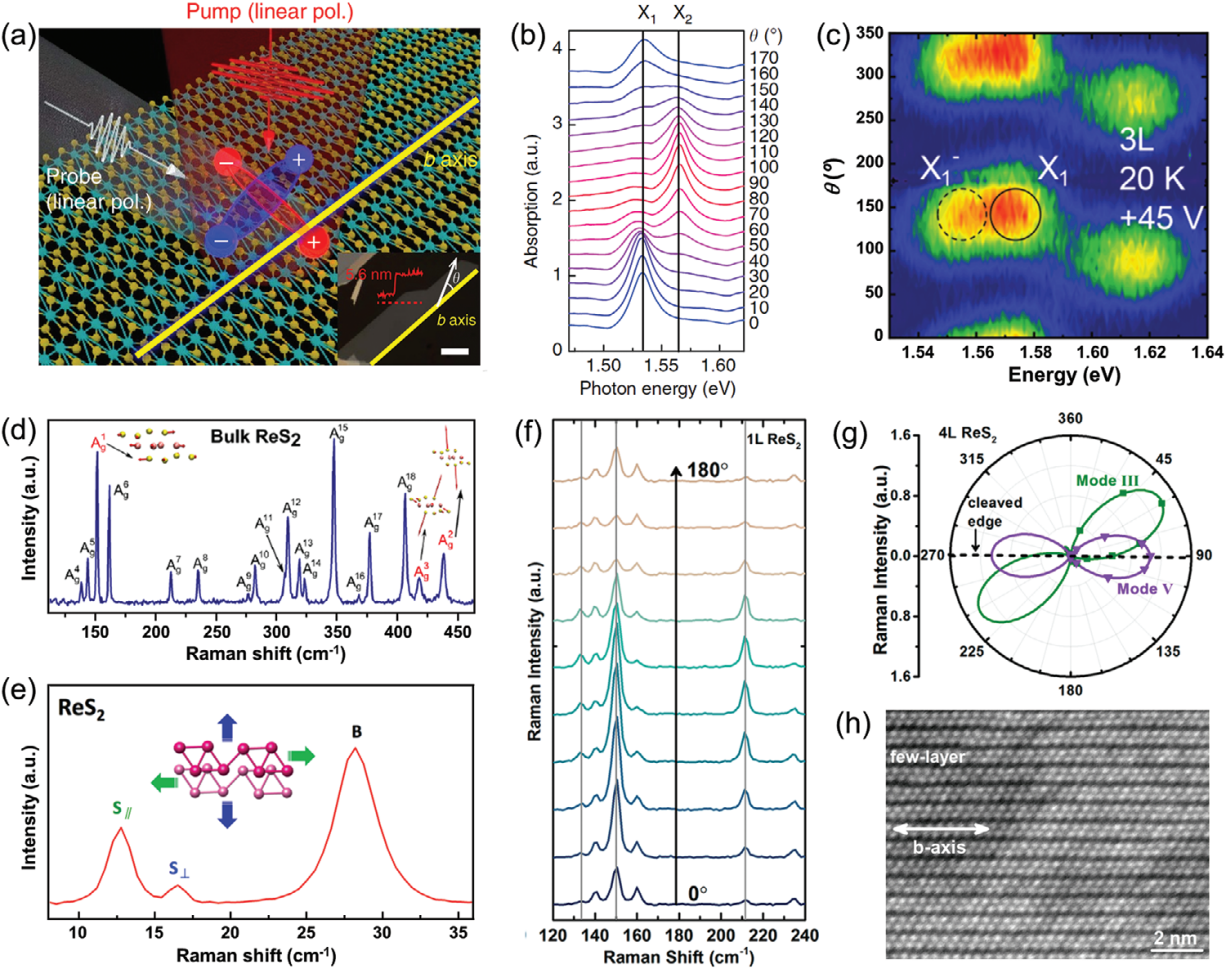
a) Schematic illustrating the pump‐probe experiment of few‐layer ReS_2_. Two electron(−)‐hole (+) pairs represent anisotropic excitons, X_1_ (blue) and X_2_ (red). Inset is optical image of few‐layer ReS_2_. b) Polarization‐dependent absorption spectra of few‐layer ReS_2_. Reproduced with permission.^[^ ^14^ ^]^ Copyright 2016, Springer Nature. c) 2D contour plot of the PL spectra as a function of the polarization angle *θ* measured at 20 K at an applied gate voltage of +45 V. Reproduced with permission.^[^ ^59^ ^]^ Copyright 2019, Wiley‐VCH. d) Raman spectrum of bulk ReS_2_ taken with 515 nm laser excitation. Reproduced with permission.^[^ ^66^ ^]^ Copyright 2017, American Chemical Society. e) Ultralow frequency Raman spectra of 2‐layer ReS_2_. Reproduced with permission.^[^ ^68^ ^]^ Copyright 2016, American Chemical Society. f) Polarized Raman spectra of ReS_2_ measured under different sample rotation angle *θ*. g) Polar plots of the peak intensity for mode III (152 cm^−1^) and mode V (213 cm^−1^) as a function of *θ*. h) High‐magnification STEM image of the corresponding ReS_2_ sample. The write arrow indicates the *b*‐axis (R4‐chain) direction. Reproduced with permission.^[^ ^16^ ^]^ Copyright 2015, American Chemical Society.

It is worth noting that the low symmetry structure of ReX_2_ leads to the emission of excitons exhibit interesting linear dichroism,^[^ ^14^, ^17^, ^59^, ^60^, ^61^ ^]^ as illustrated by the blue and red electron–hole pairs in **Figure** **3**a.^[^ ^14^ ^]^ The nondegenerate absorption peak of X_1_ (1.58 eV) and X_2_ (1.63 eV) originate from the recombination of neutral exciton show strong polarized dependence (Figure 3b). Especially, the optical Stark shift of two energetically nondegenerate exciton states can be selectively tuned by manipulating light polarization. Moreover, the emission of the negative trions X_1_^−^ (1.56 eV) and X_2_^−^ (1.62 eV) also exhibit a distinct polarization‐dependent characteristic (Figure 3c).^[^ ^18^ ^]^ An interesting ultrafast exciton quantum beats was observed in the atomically thin ReS_2_, which can be further modulated by tuning the laser polarization. In this regard, the highly anisotropic excitons and trions in ReX_2_ provide great feasibility for polarization‐sensitive applications, such as anisotropic ultrafast optics and optical logic circuits.

As a nondestructive and powerful technique for characterizing the Brillouin‐zone center (Γ‐point) phonon properties of materials, Raman spectroscopy provides information about the structure, lattice symmetry, crystal quality, thickness, defects, and dopants.^[^ ^62^, ^63^, ^64^, ^65^ ^]^ Figure 3d shows the high frequency Raman spectra of bulk ReS_2_ crystal, 18 first‐order intralayer modes can be clearly observed within 100–450 cm^−1^, which belong to A_g_ symmetry and named as A_g_^1^−A_g_^18^ modes.^[^ ^66^ ^]^ For convenience, Roman numerals I, II, III, IV… are utilized to represent the 18 Raman modes of ReS_2_, in which three primary intralayer Raman modes III, IV and V are located at 152, 162, and 213 cm^−1^, respectively. Notably, the frequency shift of each Raman mode shows tiny variation from monolayer to bulk ReS_2_, which is different from the strong thickness‐dependent Raman spectra variation of most of 2D layered materials and is attributed to the weak interlayer coupling.^[^ ^67^ ^]^ Moreover, ReSe_2_ also has abundant Raman vibration modes with negligible layer number dependence because of its structural similarity with ReS_2_.^[^ ^46^, ^67^ ^]^ In this respect, the high frequency Raman spectra of ReX_2_ cannot be utilized to identify their layer numbers as that used in other 2D materials.

The weak van der Waals (vdW) coupling in 2D layered materials usually leads to the emergence of interlayer phonon modes which are typically located in ultralow frequency regime.^[^ ^67^, ^68^, ^69^ ^]^ Hence, the ultralow frequency Raman spectra can offer valuable information about the interlayer charge exchanges, screenings, scatterings and their stacking order, and thus renders that a favorable sign for determining the thickness of the 2D material. Figure 3e displays the ultralow frequency Raman modes (0–35 cm^−1^) of 2‐layer ReS_2_, which belong to the layer shear modes (S) and layer breathing modes (B).^[^ ^68^ ^]^ For the S modes, the layers oscillate rigidly against each other in the crystal plane, while for the B mode (28 cm^−1^), the oscillation amplitude is perpendicular to the layer plane. Unlike the high symmetry TMDs such as MoS_2_, where S modes are doubly degenerate and have the same frequency,^[^ ^70^, ^71^ ^]^ the S modes of ReS_2_ are nondegenerate due to the low crystal symmetry, and thus two types of S modes (S_∥_ located at 13.0 cm^−1^ and S_⊥_ located at 16.5 cm^−1^) are clearly resolved in the Raman spectra. Furthermore, the frequency of all the observed S and B modes of ReS_2_ and ReSe_2_ decrease with the layer number increases, and ultimately merge into the Rayleigh background when the thickness is more than ten layers.^[^ ^67^ ^]^ The peak position of the low frequency Raman modes have a linear relationship with the number of layers, providing an effective method to identify the thickness of ReX_2_. In particular, the low frequency Raman spectra can also be used to judge the stacking order in ReX_2_ crystal, as the interlayer phonon modes are sensitive to lattice coupling between the ReX_2_ layers.^[^ ^68^, ^69^, ^72^ ^]^ Additionally, from the aspect of force constants per unit (*k*_S_) of low frequency modes, the *k*_S_ of ReS_2_ and ReSe_2_ are 17.1 and 17.1 × 10^18^ N m^−3^, much smaller than the force constant of other materials (MoS_2_: 25 × 10^18^ N m^−3^, MoTe_2_: 34 × 10^18^ N m^−3^, and PtSe_2_: 46 × 10^18^ N m^−3^, etc.).^[^ ^55^, ^67^, ^70^, ^73^ ^]^ Above results indicate that ReX_2_ has a rather weak interlayer coupling.

As low symmetry 2D material, the phonon coupling of ReX_2_ shows strong anisotropic properties, which has been confirmed via angle‐resolved polarized Raman spectra.^[^ ^16^, ^46^, ^68^, ^69^, ^74^ ^]^ Figure 3f shows the polarized Raman spectra of monolayer ReS_2_ as a function of sample rotation angle *θ*.^[^ ^16^ ^]^ The intensity of each Raman peak varies strongly with the change of *θ*, while the peak positions remain unchanged, indicating strong 2D in‐plane anisotropic properties. Figure 3g presents the polar plots of the peak intensity of mode III (152 cm^−1^) and mode V (213 cm^−1^) as a function of *θ*. The direction of the maximum of the Raman tensor for the mode V is coincident with the direction of the *b* axis (Figure 3h), which can be utilized as a convenient optical method to determine the crystallographic orientation of ReS_2_. More than that, the intensity of both S and B modes in the ultralow frequency Raman of ReS_2_ show strong polarization dependence as well, which can also be used to identify the in‐plane crystal orientation of ReX_2_.^[^ ^68^, ^69^ ^]^

In view of 1T′ ReX_2_ belongs to the *Ci* space group, its structure has only one inversion center. Hart et al. found turning a layer upside‐down is not a symmetry operation, but reverses the sign of the angle between the two nonequivalent in‐plane crystallographic axes.^[^ ^75^ ^]^ They produced few‐layer ReS_2_ (and ReSe_2_) samples with controlled “up” or “down” orientations by micromechanical cleavage and utilized the angle‐resolved polarized Raman microscopy to distinguish these two orientations. The angles between the maximum of mode III and mode IV and mode V in the polar plots are opposite for the two ReS_2_ flakes, indicating the distinct vertical orientations, “upward” and “downward.” In reality, this is a very important discovery because different vertical orientation may lead to a new source of domain structures, especially for the large area growth of ReX_2_ by CVD or other similar methods. All of the above research works establish angle‐resolved polarized Raman spectra as an effective approach for the identification of both in‐plane and vertical orientation of ReX_2_.

Besides, Zhao and coworkers reported an unexpected layer‐dependent, strong, and anisotropic second harmonic generations (SHGs) in atomically thin ReS_2_.^[^ ^76^ ^]^ Appreciable (negligible) SHGs are obtained from even (odd) numbers of ReS_2_ layers, which is opposite to the layer dependence of SHGs obtained in group‐VI TMDs. The SHG signals of ReS_2_ show strong anisotropy, indicating its distorted lattice structure with more unequal and nonzero second‐order susceptibility elements.

## 3 Preparation and Structure Modulation of ReX_2_

The controlled preparation of 2D ReX_2_ with desirable size, morphology, thickness, and crystal quality is of significant importance for the investigation of their optical, electronic and thermal properties, as well as the exploration of their potential applications. In this section, we first summarize the synthetic methods of ReX_2_, which can be categorized into two parts: i) top‐down production method and ii) bottom‐up production method. The top‐down production methods include mechanical exfoliation and liquid‐phase exfoliation. The bottom‐up production methods incorporate chemical vapor transport (CVT), physical vapor deposition (PVD) and CVD. On account of the high productivity and better controllability of the CVD method, we will give more emphasis on the CVD synthesis strategies of ReX_2_. Not only that, we also discuss the advantages and limitations of each approach and propose some future potential research directions for the improvement of ReX_2_ synthesis methods. Finally, exploration of the unusual structural features and growth mechanism of ReX_2_ as well as their structure modulation (such as constructing Re‐based 2D alloys and heterostructures) are introduced.

### 3.1 Chemical Vapor Transport

**Figure 4:**
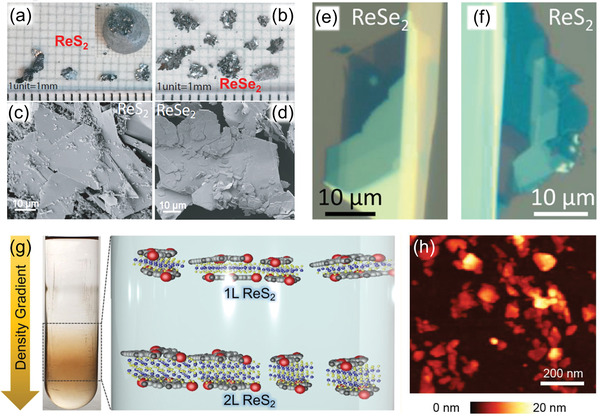
Photograph of the CVT‐grown bulk a) ReS_2_ (inset shows the ingot as removed from the quartz tube) and b) ReSe_2_ crystals. SEM images of c) ReS_2_ and d) ReSe_2_, showing the surface morphology of the flakes. Reproduced with permission.^[^ ^80^ ^]^ Copyright 2016, American Chemical Society. Optical images of mechanical exfoliated monolayer and few‐layer e) ReS_2_ and f) ReS_2_ nanosheets. Reproduced with permission.^[^ ^67^ ^]^ Copyright 2016, American Chemical Society. g) Photograph and schematic diagram of ReS_2_ dispersions prepared by the density gradient ultracentrifugation. h) AFM image of solution‐processed ReS_2_ nanosheets following deposition on a Si wafer. Reproduced with permission.^[^ ^87^ ^]^ Copyright 2016, American Chemical Society.

Chemical vapor transport (CVT) growth is a classic and mature technique for the synthesis of bulk crystal materials.^[^ ^77^ ^]^ At present, the fundamental property investigation and functional device fabrication of the most 2D materials are highly relied on the 2D nanosheets mechanically exfoliated from the bulk crystals synthesized via CVT method,^[^ ^78^, ^79^ ^]^ and ReX_2_ is no exception. In general, the CVT method is equipped with a sealed ampoule tube containing the precursor and transport agent, and then the growth is performed in low pressure and high temperature for about several to ten days.^[^ ^77^ ^]^ Bhattacharya and coworkers developed a modified Bridgman method without transport agent to synthesize high‐quality ReS_2_ and ReSe_2_ bulk single crystals (**Figure** **4a**–**d**) by using the pure Re powder and S/Se powder.^[^ ^80^ ^]^ Just recently, Jiao and coworkers developed an approach for directly synthesizing thin ReX_2_ flakes by CVT through carefully tuning the growth kinetics together with a specially designed quartz ampoule with a neck to separate the powders from the growth substrate.^[^ ^81^ ^]^ With this approach, few‐layer and even monolayer ReSe_2_ nanosheets with high quality were grown on mica or sapphire substrate. Hence, CVT is an ideal approach for preparing the high crystallinity single crystal ReX_2_ bulk and thin flakes. Nevertheless, the time‐consuming, strict and harsh reaction conditions, as well as uncontrollable morphology, size and thickness limit its widespread exploitation, especially regarding the preparation of large‐area monolayer material for device applications.

### 3.2 Mechanical Exfoliation

Since the discovery of graphene, mechanical exfoliation of 2D layered materials from bulk parent materials has been the optimal preparation method for investigating their intrinsic physical and chemical properties.^[^ ^78^, ^82^, ^83^ ^]^ The main step of this top‐down method is illustrated as follows: first the single crystal bulk material is attached to the adhesive on the Scotch tape and then another piece of tape is placed on the other side of the material, following by peeling off the two pieces of tape for several times; the freshly cleaved thin flake on the Scotch tape is then attached to a clean, flat substrate (generally 300 nm SiO_2_/Si substrate); finally few layers or monolayer material can be obtained on the target substrate. Actually, mechanical cleavage has been widely used as a facile technique to obtain few‐layer ReX_2_ with high quality, clean surface, and relatively large area for studying their fundamental properties and potential applications.^[^ ^13^, ^19^, ^84^ ^]^ Figure 4e,f shows the typical OM images of ReSe_2_ and ReS_2_ on SiO_2_/Si substrate with different layers exfoliated from their bulk materials.^[^ ^67^ ^]^ However, this method still has many shortcomings such as lack of control over thickness, randomness in size and shape, as well as low productivity. An improved exfoliated method needs to be explored toward high yield, large area and easy control with good repeatability for preparing desirable ReX_2_ film in the future.

### 3.3 Liquid‐Phase Exfoliation

Liquid‐phase exfoliation is a powerful method for the scaled‐up fabrication of ultrathin nanosheets form bulk layered materials. Dispersing them in the solvent is a direct and effective way to reduce the interlayer van der Waals force, which allows the liquid‐phase exfoliation method possible to achieve industrialization and commercialization due to its high‐yield mass production.^[^ ^85^, ^86^ ^]^ At present, the liquid‐phase exfoliation method of ReX_2_ materials can be divided into two primary types: sonication‐assisted exfoliation and ion intercalation exfoliation.^[^ ^87^ ^]^ Hersam and coworkers reported the exfoliation and layer‐by‐layer isopycnic density gradient ultracentrifugation sorting of high‐density ReS_2_ nanosheets in aqueous surfactant solutions (Figure 4g).^[^ ^87^ ^]^ First, ReS_2_ powder was ultrasonically exfoliated in deionized water with the amphiphilic small‐molecule surfactant sodium cholate. Then, the ReS_2_ dispersion was purified by using centrifugal separation at 7500 rmp aims to remove the unexfoliated ReS_2_ flakes. The supernatant was subsequently collected and further ultracentrifuged at 20 000 rpm to precipitate large‐size ReS_2_ nanosheets. Finally, the relatively uniform ReS_2_ nanosheets with average thickness of ≈3 nm and lateral size of 50–100 nm were achieved as confirmed by AFM (Figure 4h). Additionally, based on the Hansen solubility theory, a mixed‐solvent strategy was developed for the facile and green preparation of few‐layered ReS_2_ nanosheets by exfoliating bulk ReS_2_ in an ethanol‐water mixture.^[^ ^88^ ^]^ It was found that 28% ethanol in 72% deionized water is the best mixture solvent for the efficient exfoliation of ReS_2_. Combining the mixture solvent and sonication assistance, large‐scale ReS_2_ nanosheets with an average lateral size of 2.3 nm and a thickness of 50–80 nm were obtained. Furthermore, ion intercalation exfoliation is another effective method for preparing ReS_2_ nanosheets, as the cation (e.g., Li^+^, Na^+^, K^+^) with small ionic radius can easily insert into the interspace of layered bulk crystals, which can significantly expand the interspace and weaken the van der Waals interaction between adjacent layers. A solvent‐free approach involving the reaction of ReS_2_ powder with lithium borohydride (LiBH_4_) was developed to effectively exfoliate ReS_2_ nanosheets, which could replace the conventional protocol involving a butyl lithium solution.^[^ ^89^ ^]^

The aforementioned methods demonstrate that liquid‐phase exfoliation is an effective approach for exfoliating ReX_2_ nanosheets in large quantities with crystal quality comparable to mechanically exfoliated ReX_2_. Nonetheless, the resulting ReX_2_ nanosheets are polydisperse with respect to lateral size and thickness, and it is challenging to achieve a uniform monolayer ReX_2_ film with large size. Additionally, some reagents and solvents may contaminate the product. In this regard, the ReX_2_ nanosheets prepared by liquid‐phase exfoliation is more suitable for energy conversion and biological applications.

### 3.4 Physical Vapor Deposition

Physical vapor deposition (PVD) is a typical bottom‐up method for the preparation of 2D nanosheets. PVD growth is an atomistic deposition process in which the solid or liquid precursors evaporate in the form of atoms or molecules, and then transports through a high vacuum or low pressure gas (or plasma) environment to the target substrate where it condenses or deposits.^[^ ^90^, ^91^ ^]^ Take the PVD growth of ReS_2_ as an example, pure ReS_2_ powder was first placed at the center of quartz tube, and then the target SiO_2_/Si substrate with facing up was placed at downstream far from the ReS_2_ powder.^[^ ^92^ ^]^ The growth was conducted at temperature of 900 °C with 50 sccm Ar as carrier gas for 1 h. Then, the as‐grown ReS_2_ sample was annealed in a sulfur‐rich environment to further improve its crystalline quality. Using this approach, centimeter‐size ReS_2_ film can be grown on SiO_2_/Si substrate, and the AFM results indicate that the thickness is about 2.3 nm corresponding to three layers ReS_2_ film. Hence, PVD growth is an effective method for preparing large‐area high‐quality ReX_2_ film. However, this method may not be versatile due to the higher melting point of the precursor material and higher vacuum requirements.

### 3.5 Chemical Vapor Deposition

Chemical vapor deposition (CVD) is one of the representative bottom‐up approaches for fabricating traditional semiconductor films, and has also been widely utilized to prepare 2D materials.^[^ ^3^, ^93^, ^94^, ^95^, ^96^ ^]^ The CVD process involves depositing solid material from gaseous phase, which is achieved by means of a chemical reaction between volatile precursors. As the gas precursors pass over the surface of the heated substrate, the resulting chemical reaction forms a solid phase and deposits onto the substrate via nucleation and following epitaxy growth. In this process, several parameters must be controlled well, like growth temperature, gas flow rate, substrate type, precursor selection, etc. Hence, as a conventional and versatile approach, CVD growth plays a critical role in the preparation of scalable and high‐quality 2D ReX_2_ film for device applications.^[^ ^97^, ^98^, ^99^, ^100^, ^101^, ^102^, ^103^ ^]^ Meanwhile, the easy command of CVD process facilitates the construction of ReX_2_‐based alloys and heterostructures for tuning their optical, electrical and magnetic properties.

Owing to the 1T′ structure and weak interlayer coupling, ReX_2_ has been found to show many unusual growth behaviors, such as off‐symmetry growth, out‐of‐plane growth and nano‐assembly growth.^[^ ^38^, ^97^, ^102^ ^]^ As a result, the morphology of CVD‐grown ReX_2_ are mostly thick flakes, dendritic shape or 3D flower‐like structures, especially large amounts of subdomains and grain boundaries exist in the ReX_2_ nanosheets. These diverse morphologies and structures of ReX_2_ together with the strong anisotropic properties and weak interlayer coupling endow them great application potential in wide fields. For example, the planar ReX_2_ nanosheets have been utilized to construct diverse electronic and optoelectronic devices. The 3D ReX_2_ nanoarrays display excellent application prospects in catalysis, lithium ion battery and sensors where abundant exposed active edges are needed. In this section, we summarized the progress of CVD growth of ReX_2_ in three aspects: the growth of planar ReX_2_ nanosheets, the exploration of the formation of subdomain and grain boundary in ReX_2_, and the growth of vertically oriented 3D ReX_2_ nanoarrays.

#### 3.5.1 CVD Growth of Planar ReX_2_ Nanosheets

**Figure 5:**
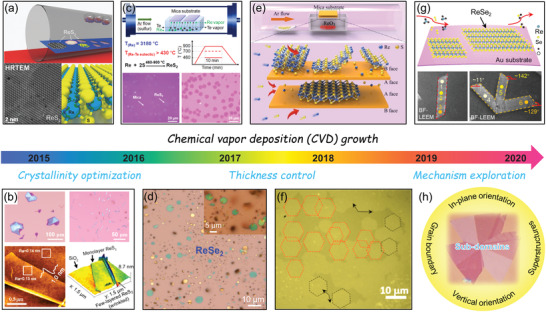
Development process of the preparation of ReX_2_ material by CVD growth. a) Schematic diagram of CVD growth of ReS_2_ using NH_4_ReO_4_ as Re precursor, and the corresponding HRTEM image. Reproduced with permission.^[^ ^97^ ^]^ Copyright 2015, Wiley‐VCH. b) Optical and AFM images showing the morphologies and thickness of ReS_2_ grown using pure Re powder as rhenium precursor. Reproduced with permission.^[^ ^98^ ^]^ Copyright 2015, Wiley‐VCH. c) Schematic diagram of the tellurium‐assisted epitaxy growth of ReS_2_, and the corresponding OM images of monolayer on mica and SiO_2_/Si substrates. Reproduced with permission. Copyright 2016, Wiley‐VCH. d) OM image of the ReSe_2_ hexagonal flakes grown on SiO_2_/Si substrates with ReO_3_ as Re precursor, inset shows the magnified OM image for the clarity. Reproduced with permission.^[^ ^101^ ^]^ Copyright 2016, Wiley‐VCH. e) Schematic diagram of the space‐confined CVD growth of monolayer ReS_2_ on mica substrate. Reproduced with permission.^[^ ^102^ ^]^ Copyright 2016, Royal Society of Chemistry. f) Optical image of CVD‐grown monolayer ReS_2_ on c‐cut sapphire substrate. Reproduced with permission.^[^ ^103^ ^]^ Copyright 2016, American Chemical Society. g) Schematic diagram of the CVD growth of monolayer ReSe_2_ on Au foil substrate, and the corresponding low‐energy electron microscopy images. Reproduced with permission.^[^ ^109^ ^]^ Copyright 2018, Springer Nature. h) Optical image showing the subdomains and grain boundaries in CVD‐grown monolayer ReS_2_ domain. Reproduced with permission.^[^ ^38^ ^]^ Copyright 2019, Wiley‐VCH.

Ajayan and coworkers reported the first synthesis of monolayer ReS_2_ nanosheets on SiO_2_/Si substrate using CVD growth (**Figure** **5**a) with ammonium perrhenate (NH_4_ReO_4_) and sulfur as Re and S precursors, respectively.^[^ ^97^ ^]^ The size of as‐grown ReS_2_ nanosheets varies from 2 to 60 µm at different growth conditions. When the growth temperature is 400–450 °C, the morphology of ReS_2_ grains is mainly hexagon with an average thickness of 13 nm. With the growth temperature rises to 450–500 °C, rounded ReS_2_ grains with the thickness of 110 nm are obtained. Notably, all the ReS_2_ grains grown at different temperatures show dendritic edges, which are attributed to the off‐symmetry anisotropic growth of ReS_2_. In addition, the Z‐contrast scanning transmission electron microscopy (STEM) image distinctly resolves the 1T′ structure of ReS_2_ with Re4‐chains are clearly visible (Figure 5a). However, a few defects can also be identified in the HRTEM image, which may be due to the excessive by‐products like SO_2_ especially NH_3_ produced during the growth process, as shown in the reaction equation below

(1) 4NH4ReO4+15S→4ReS2+7SO2+4NH3+2H2O

For improving the crystal quality of CVD‐grown ReS_2_, another early research work reported the CVD synthesis of ReS_2_ using pure element Re powder as precursor, as shown in Figure 5b.^[^ ^98^ ^]^ The growth was performed at the temperature of 750 °C under atmospheric pressure condition. Few‐layer ReS_2_ flakes and 1D nanoribbons were obtained on Si/SiO_2_ substrate as shown in the OM images. Furthermore, the HRTEM image shows that the crystal quality of ReS_2_ is higher than that prepared with NH_4_ReO_4_ as Re precursor, indicating the role of pure element Re in producing high crystallinity ReS_2_, as shown in the reaction equation below

(2) Re+2S→ReS2

However, there are only a few ReS_2_ grains scattered on the substrate, indicating quite low growth efficiency. This is reasonable, given that the much high melting point of Re (3180 °C) makes its vapor pressure in the growth system quite low at the general CVD growth temperature (<1000 °C), which greatly decreases the efficiency of nuclear and epitaxial growth of ReS_2_.

To solve the above problems, our group developed a tellurium (Te)‐assisted epitaxial growth method to synthesize large‐area, highly crystalline ReS_2_ with uniform monolayer thickness on mica substrate (Figure 5c).^[^ ^99^ ^]^ The inspired strategy derived from the Re‐Te binary eutectic, whose eutectic point can be reduced to 850 °C, and even to 430 °C when the weight ratio of Te and Re reach to 90%. With this approach, large‐scale, highly crystalline monolayer ReS_2_ grains with a lateral size of ≈20 µm were synthesized at growth temperature range from 500 to 900 °C. These results show that the growth efficiency was largely increased by introducing Te powder into the growth system, because it lowers the originally high melting point of Re and thus increases the vapor pressure of the Re precursor in the growth system. Furthermore, the morphology of ReS_2_ changes from round at 500 °C to hexagon at 600 °C, and then to serration at 700 °C, finally to dendritic structure at 850 °C. The dendritic morphology of ReS_2_ indicates an off‐symmetry growth which is attributed to the anisotropic interfacial energy induced by 1T′ structure. This work opens up new avenues for the synthesis of other 2D materials where the precursor has a high‐melting point.

At the same time, Zhai and coworkers reported the CVD synthesis of ReS_2_ on sapphire substrate using ReO_3_ as Re precursor.^[^ ^100^ ^]^ The chemical reaction is illustrated as follows

(3) 2ReO3+7S→2ReS2+3SO2

Using this improved growth approach, large‐scale hexagonal ReS_2_ flakes and continuous bilayers ReS_2_ film were successfully synthesized. HRTEM and AFM reveal that the hexagonal flakes are layered stacking crystals, while the continuous film is bilayer polycrystal. Combine with theory calculation, they found that the formation of hexagon ReS_2_ grain takes place only when the growth rate on the (100) plane is comparable to the growth rate on the (020) plane. Moreover, the partial pressure of ReS_2_ species plays a key role in the growth rate of the ReS_2_, as larger partial pressure may increase the growth rate and enhance the nucleation density at the same time. As a result, under higher partial pressure, small‐sized ReS_2_ grains nucleate simultaneously and connect to each other forming a continuous polycrystalline bilayer ReS_2_ film. Besides the synthesis of ReS_2_, Zhai and coworkers also reported the first CVD growth of ReSe_2_ by using ReO_3_ as Re precursor.^[^ ^101^ ^]^ Large‐scale hexagonal‐shape ReSe_2_ flakes with thickness of ≈4.2 nm and lateral size of ≈5 µm were grown on SiO_2/_Si substrate (Figure 5d). The perfect lattice structure shown in HRTEM image evidence the high‐quality of CVD‐grown ReSe_2_ flakes. Above results demonstrate that ReO_3_ is an ideal Re precursor for the CVD synthesis of large‐scale, high crystallinity ReS_2_ and ReSe_2_ materials.

Though tremendous progresses have been achieved on the synthesis of ReX_2_, the high volatility and versatile valence states of Re oxides make the controllable synthesis of high‐quality ReX_2_ film with large grain size, planar and uniform monolayer thickness quite difficult. In view of these problems, our group conducted an in‐depth study on the decomposition process of ReO_3_ powder at different temperatures through thermogravimetric analysis.^[^ ^102^ ^]^ It was found that the unstable ReO_3_ can quickly decompose into Re_2_O_7_ (which sublimes easily) and ReO_2_ (which is less volatile) via a disproportionation reaction (reaction 4) when the temperature rises to 400 °C. Thus, the detailed reaction process for the growth of ReS_2_ via ReO_3_ is shown below

(4) 3ReO3→Re2O7+ReO2

(5) 2Re2O7+15S→4ReS2+7SO2

The high volatility of Re_2_O_7_ (melting point: 220 °C and boiling point: 360 °C) brings abundant Re precursor vapor and nucleation sites during the growth system, which leads to the growth of ReS_2_ uncontrollable, and thus large amount of thick flakes, polycrystal films, and even 3D flower‐like structures are grown on substrate.

Based on the above understanding, a space‐confined CVD growth approach was developed to synthesize large‐area and consecutive monolayer ReX_2_ film on mica substrate.^[^ ^102^, ^104^ ^]^ Figure 5e illustrated the schematic diagram of the space‐confined CVD growth approach. In brief, two pieces of freshly exfoliated mica were stacked together and then placed on the top of ceramic boat containing ReO_3_ powder. The electrostatic attraction between two stacked micas forms a microreaction space which greatly decreases the precursors vapor and thus reduces the nuclear density and growth rate of ReS_2_. As a result, large‐scale monolayer ReS_2_ hexagonal domains with size up to 60 µm were grown on the inside of the stacked mica, and even continuous uniform monolayer ReS_2_ film can be obtained by extending the growth time. Owing to the simplicity and effectivity, the space‐confined CVD growth approach has been widely utilized to controllably synthesize large‐area monolayer ReSe_2_ film and other 2D TMDs materials.^[^ ^104^, ^105^, ^106^, ^107^ ^]^

Besides the above works, Zhang's group reported the synthesis of ReSe_2_ with high crystal quality on gold foil substrate, which provides the convenience for in‐situ studying their electrical properties by using scanning tunneling microscope (STM).^[^ ^108^, ^109^, ^110^, ^111^ ^]^ Lee and coworkers prepared high uniformity and continuity ReS_2_ film on the transparent flexible glasses at a relatively low temperature.^[^ ^112^ ^]^ Combining the tellurium (Te)‐assisted epitaxial growth with another type of space‐confined CVD configuration where an inner tube was placed in the reactor, Li and coworkers realized the growth of substrate‐scale continuous atomically thin ReS_2_ film with uniform monolayer thickness on mica substrate.^[^ ^113^ ^]^ Furthermore, Zhen and coworkers prepared large‐area continuous ReS_2_ films by CVD growth on mica substrate with the mixture of metal Re and Re_2_O_7_ as Re precursor.^[^ ^114^ ^]^ After achieving the controlled synthesis of large‐area monolayer ReX_2_ film, researchers begin to explore its unusual structure, growth behavior and mechanism, as well as to clarify the relationship between them (Figure 5f–h). In view of the importance of this part, we will give it a special and systematic discussion in the following section.

All in all, CVD growth is the most convenient and effective method for the controlled preparation of 2D Re‐based materials. An obvious advantage of this method, in addition to its relative simplicity, is the possibility of growing large‐scale ReX_2_ films with their lateral sizes only limited by the size of the substrate used. However, it is still challenging to precisely control over the thickness and uniformity of the ReX_2_ film due to the point diffusion volatilization of precursor powder. In this regard, an improved CVD method like metal‐organic chemical vapor deposition (MOCVD) which uses gaseous precursors may be an effective method to solve the above problems, because the growth process can be well controlled by regulating the flow rate of gas precursors just as the CVD growth of graphene. Therefore, the research in the synthesis of 2D ReX_2_ materials is still in the infant stage. There are still greater opportunities for research innovations in developing new synthesis approaches with the goal of achieving high quality and large‐scale ReX_2_ materials at relatively low cost and better controllability.

#### 3.5.2 Subdomains and Grain Boundaries in ReX_2_

As is well known, the CVD growth of 2D TMDs experiences the nucleation of grain and the subsequent grain merger until they grow into large‐area films.^[^ ^3^, ^115^ ^]^ During this process, grain boundaries (GBs) are inevitably formed between adjacent grains. ReX_2_ naturally cannot avoid the arise of GBs during present CVD growth processes and technical means. However, different from the high‐symmetry 2H TMDs (such as MoS_2_ and WS_2_), the low‐symmetry structure of ReX_2_ endows them more degree of freedom during the growth process and thus bring more complex GBs. In the past several years, along with the discovery of a series of new GB structures, many abnormal growth behaviors, morphology and structure features of ReX_2_ have been gradually uncovered. In this section, we mainly introduce the discovery of subdomains and GBs in CVD‐grown ReX_2_ and the exploration of growth mechanism of unusual multi‐domain structures, which are critical to further control its preparation toward large‐scale device applications.

**Figure 6:**
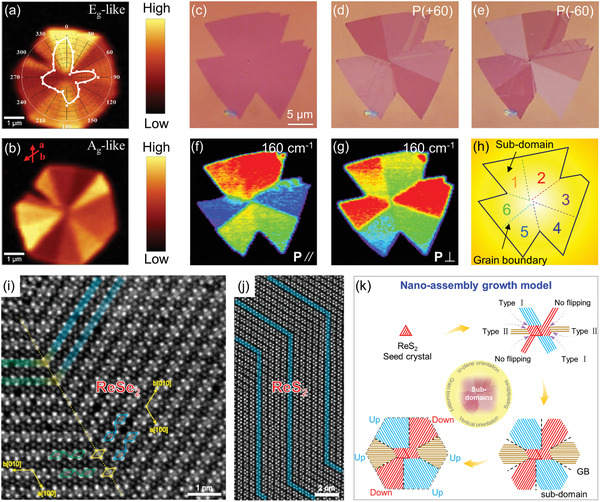
Raman intensity mapping of a) in‐plane E_g_‐like and b) out‐of‐plane A_g_‐like modes of CVD‐grown ReSe_2_ flakes, the variation in the integrated intensities of these two modes with the incident light polarization are overlapped with the respective Raman mapping image. Reproduced with permission.^[^ ^101^ ^]^ Copyright 2016, Wiley‐VCH. c) OM image of CVD‐grown monolayer ReS_2_ domain, and the corresponding ARPOM images taken at the angle of d) +60° and e) −60° between polarizer and analyzer. f,g) Polarized Raman mapping images of the ReS_2_ domain for the vibrational modes at 160 cm^−1^ under parallel (P//) and perpendicular (P_┴_) polarization configurations. h) Schematic structure of the ReS_2_ composed of several subdomains and grain boundaries. Reproduced with permission.^[^ ^38^ ^]^ Copyright 2019, Wiley‐VCH. i) The ADF‐STEM image of ReSe_2_. Two domains with different orientations are separated by a twin boundary, which are marked in blue, green, and yellow line, respectively. j) The ADF‐STEM image of ReS_2_ with divergent diamond chain orientations. Reproduced with permission.^[^ ^15^ ^]^ Copyright 2015, American Chemical Society. k) Schematic diagram of the "nanoassembly growth model" of ReS_2_ showing the formation process of subdomains and GBs. Reproduced with permission.^[^ ^38^ ^]^ Copyright 2019, Wiley‐VCH.

In the earliest work about CVD growth of ultrathin hexagonal ReSe_2_ flakes carried out by Zhai's group,^[^ ^101^ ^]^ they performed the polarized Raman mapping of ReSe_2_ to confirm the dependence of Raman vibration modes on the *b*‐axis orientation. Surprisingly, the Raman intensity of E_g_‐like (**Figure** **6**a) and A_g_‐like (Figure 6b) modes vary at different areas and show distinct polarization dependence. Accordingly, they deduced that this might be due to the grain orientation alteration in the same single ReSe_2_ grain. This is the first time researchers have realized that the hexagonal ReSe_2_ grain should not be single crystal but polycrystalline. To visualize the possible existed GBs, they heated the sample at about 300 °C in the air just as the visualization method of graphene grown on copper substrate. After heat treatment, the GBs can be observed clearly and a complete ReSe_2_ flake is divided into six parts, confirming the existence of GBs in the ReSe_2_ grain. Tongay and coworkers reported the CVD synthesis of ReS_2_ monolayer on c‐cut sapphire substrate and got insight into the multidomain architectures in the CVD‐grown ReS_2_.^[^ ^103^ ^]^ Angle‐resolved polarized Raman spectroscopy (ARPRM) measurements demonstrate the presence of structurally anisotropic ReS_2_ subdomains in the CVD‐grown ReS_2_ triangle or hexagon domain. Furthermore, our group developed an angle‐resolved polarized optical microscopy (ARPOM) imaging technique to directly visualize the subdomains and GBs in CVD‐grown monolayer ReS_2_ (Figure 6c–e).^[^ ^38^ ^]^ The OM image of CVD‐grown monolayer ReS_2_ domain shows it seems to be a single crystal, while six subdomains and the corresponding GBs were clearly distinguished in the same ReS_2_ domain from their ARPOM images. Similar multi‐domain structures were also observed from polarized Raman mapping of the two primary vibration modes at 160 and 211 cm^−1^ measured under parallel‐polarized (P_//_) and cross‐polarized (P_┴_) configurations of incident and collection lights (Figure 6f,g). These results demonstrate that the CVD‐grown ReS_2_ domain is composed of several subdomains with different lattice orientations, and GBs are formed between each adjacent subdomain, as schematically shown in Figure 6h. Notably, the GBs and subdomains in the ReS_2_ domain are grown from one nucleation center, which is different from that in high‐symmetry 2D materials where the GBs only originate from the splice of different domains.

Above research works convincingly confirmed the existence of GBs and subdomains in the CVD‐grown ReX_2_ domain, and preliminarily established its domain architectures, where each subdomain has different Re‐chain directions and splicing together forms GBs. In view of the novel domain architectures, researchers are interested in the atomic‐scale structure of CVD‐grown ReX_2_, especially with respect to how the subdomains and GBs are formed in space and in energy scale, which are critical to revealing the growth mechanism of ReX_2_ and tailored design of them in a controllable way. Toward that end, researchers have given a detailed study to the atomic structure features of ReS_2_ under the assistance of aberration‐corrected STEM. Suenaga and coworkers discovered two distinct Re‐chain orientations with an angle of ≈121° in mechanical exfoliated monolayer ReSe_2_ domain (Figure 6i) and ReS_2_ domain (Figure 6j).^[^ ^15^ ^]^ A mirror‐symmetric GB where the two different Re‐chains sharing the four Re atoms separates the ReX_2_ domain into two subdomains. Especially, the Re‐chain direction of monolayer ReX_2_ can be altered through in‐situ electron beam irradiation at 500 °C, indicating a Re‐chain reconstruction/flip occurs through slight displacement of Re atoms from its perfect structure. It was deduced that the Re‐chain flip can reduce the energy of the system and keep the structure become energetically favored. Importantly, the similar Re‐chain flip was commonly observed in the CVD‐grown ReS_2_ and ReSe_2_ domains,^[^ ^103^, ^109^ ^]^ indicating the possibility of Re‐chain flip inducing the formation of ReX_2_ multidomain structures. Zhang and coworkers reported the on‐site investigations of the structural and electronic properties of CVD‐grown ReSe_2_ on a conductive Au foil substrate.^[^ ^108^, ^109^ ^]^ Using scanning tunneling microscopy and spectroscopy (STM and STS), they identified the top four non‐identical Se atoms in a unit cell of the anisotropic ReSe_2_. In particular, a perfect lattice coherence and an invariable bandgap were found across the commonly observed mirror‐symmetric GBs in ReSe_2_, which considerably differ from the conventional isotropic TMDs featured with defect structures and additional states inside the bandgap. This work provides a key reference to the fundamental properties of 2D anisotropic ReX_2_ and the abnormal structural and electronic properties at the GBs, which could promote their practical applications in electronic and photonic devices as well as the energy‐related fields.

To further get insight into the nature behind the CVD growth of multidomain ReX_2_, our group reported a systematic exploration of the atomic structure, formation mechanism, and modulation strategy of GBs in the ReS_2_.^[^ ^38^ ^]^ Using aberration‐corrected STEM, we identified two major categories of GBs in one ReS_2_ domain: the joint GBs, and the Re4‐chain reconstruction‐induced GBs, which are significantly different from those observed in the high‐symmetry 2D TMDs materials. These diverse GBs imply the occurrence of different Re‐chain reconstructions during the ReS_2_ growth process, which induces subdomains with altered in‐plane or/and vertical orientations. Based on the structural features of these GBs, we proposed a novel "nanoassembly growth model" to describe the formation mechanism of ReS_2_ multi‐domain structures. First, the usually observed hexagonal ReS_2_ domain is started from a single crystal nucleus. Then, the growth moves to the Re4‐chain reconstruction stage due to the deviation of atom bonding from its equilibrium state and the strain field effect, which changes the in‐plane and vertical orientations of as‐grown ReS_2_ by 120° and 180°, respectively. During further growth process, each pair of adjacent subdomains merging together bring a particular in‐plane (60° angle) and vertical (0° or 180°) orientational relationship. Finally, a hexagonal ReS_2_ domain composed of six subdomains with different in‐plane and vertical orientations is obtained, where both the twin boundary (mainly located in the nucleation region) and the joint GB (between adjacent subdomains) are present. This growth model accounts for the abnormal growth behavior, morphology, and a variety of structural features observed in CVD‐grown ReS_2_ and is sufficiently versatile to be extended to ReSe_2_ and other low‐symmetry 2D materials. First‐principle calculations show that these GBs have several interesting properties, including new electron state and ferromagnetism, which are highly desirable for the realization of novel 2D electronics. This work highlighted here presents a clear picture about the complicated assemble process of ReX_2_ multi‐domain structure from its nucleary to growth, and provides useful guidance for further studying the low‐symmetry 2D materials. Afterward, Kang and coworkers also investigated the structure evolution of CVD‐grown hexangular ReS_2_ domains and proposed a dislocation‐involved anisotropic growth mechanism which is quite similar to the above demonstrated nano‐assembly growth mechanism.^[^ ^116^ ^]^ So far, nearly all the abnormal growth behaviors and structure features of CVD‐grown ReX_2_ have been clearly revealed. Importantly, the growth mechanism of ReX_2_ could be extended to understand and control the growth of other low‐symmetry 2D materials (such as GaTe, 1T′ MoTe_2_/WTe_2_, etc.) where similar multidomain structures were also observed.^[^ ^117^, ^118^, ^119^ ^]^

Besides, there are also many defects in the CVD‐grown ReX_2_, which affect its properties and applications. However, compared to the widely and deeply studies about defects of group‐VI TMDs (such as MoS_2_), the study about defects of Re‐based TMDs is really lack. Presently, several research works have explored the defects in ReX_2_ and their effect on its phase structure, anisotropy electrical transport and the catalysis properties.^[^ ^15^, ^103^, ^120^, ^121^, ^122^ ^]^ For instance, chalcogen‐deficiency mediated structure transformation of ReS_2_ shows that the sulfur atom loss reduces the spacing between two Re diamond chains by 20.6% from the original value (≈0.34 nm) in the *b* direction to form more constricted structure (≈0.27 nm).^[^ ^15^ ^]^ Such sulfur deficiency can also give rise to a distinct phase transformation in the a direction. This “diamond flip” transformation leads to a new phase consisting of wider double Re zigzag chain structure found in the *a* direction, which have never been observed in TMDs of group‐VI. Moreover, the commonly observed S‐Re‐S pair vacancies in CVD‐grown ReS_2_ change the atomic level interaction sufficiently that can lead to a 180° in‐plane chain rotation.^[^ ^103^ ^]^ Zhai and coworkers found the electronic anisotropy of CVD‐grown bilayer ReS_2_ can be largely adjustable, which is attributed to the angle‐dependent scattering from defects or vacancies.^[^ ^120^ ^]^ Furthermore, the abundant Re atoms vacancies with lower Gibbs free energy for H adsorption activate the inert basal plane of ReS_2_ as highly active sites for hydrogen evolution reaction.^[^ ^122^ ^]^ Notably, owing to the distorted 1T structure of ReX_2_, the six X atoms bonded to one Re atoms are inequivalent in space, and the four Re atoms in one Re4 cluster are also inequivalent. In this regard, there should be several different types of X‐defect and Re‐defect in ReX_2_, which would bring different effects to its properties and applications. Therefore, deeply study to these various types of defects from structure to property and controllably create (or repair) these diverse defects to modify (or improve) the properties of ReS_2_ are deserve to be further explored in the future.

#### 3.5.3 CVD Growth of Vertically Oriented ReX_2_ Arrays

**Figure 7:**
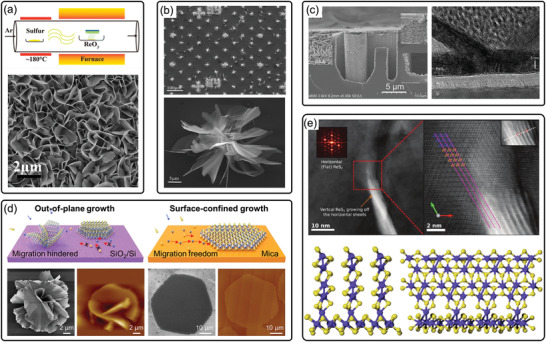
a) Schematic of ReS_2_ growth and SEM images of ReS_2_ grown on SiO_2_/Si substrate. Reproduced with permission.^[^ ^123^ ^]^ Copyright 2016, American Chemical Society. b) SEM images of ReS_2_ flowers grown on the SiO_2_ substrate and the arrays of ReS_2_ flowers fabricated on the Pt metals. Reproduced with permission.^[^ ^124^ ^]^ c) Cross‐sectional TEM image of a deposited ReS_2_ film and SEM images of the film deposited on 3D structure. Reproduced with permission.^[^ ^125^ ^]^ Copyright 2018, Wiley‐VCH. d) Schematic diagrams of the two different growth behaviors of ReS_2_ on SiO_2_/Si and mica substrates. Reproduced with permission.^[^ ^102^ ^]^ Copyright 2016, Royal Society of Chemistry. e) Atomic resolution imaging to probe the mechanism for vertical growth and a cross section of adjacent vertical sheets and a single vertical sheet attached to the horizontal sheet along a row of adjacent B‐sites. Reproduced with permission.^[^ ^127^ ^]^ Copyright 2018, Wiley‐VCH.

Taking advantage of the fact that ReX_2_ is prone to out‐of‐plane growth, several research groups have prepared a variety of vertically oriented 3D ReX_2_ arrays. Koratkar and coworkers demonstrated that with the proper precursors and appropriate tuning of the CVD growth conditions, vertically orientated ReS_2_ nanosheets can be grown on a series of substrates such as SiO_2_/Si, mica, and carbon as well as gold foil (**Figure** **7**a).^[^ ^123^ ^]^ Meanwhile, the packing density, sheet size, and crystal quality of the ReS_2_ nanosheets can be controlled by adjusting growth temperature and sulfur concentration. Wang and coworkers demonstrated a Pt metal induced nucleation to successfully realize the CVD growth of ReS_2_ flowers at controlled locations (Figure 7b).^[^ ^124^ ^]^ To achieve the large‐scale fabrication, a highly controllable and precise atomic layer deposition (ALD) technique, another bottom‐up synthesis approach, is applied to deposit 3D ReS_2_ nanoarrays.^[^ ^125^ ^]^ By controlling the growth temperature (from 120 to 500 °C), 3D ReS_2_ nanosheets with vertical orientation and petal‐like morphology (Figure 7c) were obtained on large‐area (5 cm × 5 cm) Al_2_O_3_ coated Si (110) substrate using easily sublimated ReCl_5_ and H_2_S as precursors. The developed ALD technique offers a route to upgrade the preparation of 3D ReS_2_ nanoarrays to an industrial scale. Similarly, several other research works have also prepared the 3D ReX_2_ materials with various morphologies and shapes via the CVD growth for diverse function applications.^[^ ^25^, ^126^ ^]^ Above research works demonstrate a fact that ReX_2_ exhibits much greater propensity to grow into vertical 3D nanoarrays compared to other TMDs (such as MoS_2_ or WSe_2_), which highlights the potential of the material for applications beyond planar structure architectures.

To reveal the underlying growth mechanism of vertically oriented ReX_2_, our group compared its different growth behaviors on SiO_2_/Si and mica substrates as shown in Figure 7d.^[^ ^102^ ^]^ Obviously, thick flakes and flower‐like shapes of ReS_2_ tended to grow on the SiO_2_/Si substrate, whereas uniform monolayer ReS_2_ film with large domain size and hexagonal morphology were obtained on the mica substrate. The distinct growth behaviors can be understood from the 1T′ structure of ReS_2_ itself and the surface properties of the two substrates. The rough SiO_2_ surface with abundant dangling bonds and defects brings a high energy barrier for surface migration of adatoms, which hinders the free migration of adatoms on the substrate during ReS_2_ growth. In contrast, the flat and inert surface of the mica allows the surface migration energy barrier of the adatoms much lower than that on the SiO_2_/Si substrate. Hence, the atomically flat surface of mica facilitates the migration of adatoms on it during CVD growth. In addition, the weak van der Waals interaction between the mica and atom clusters in favor of the surface‐confined growth while avoiding out‐of‐plane growth, which is critical to growing ReS_2_ film with uniform monolayer thickness. Koratkar and coworkers further investigated the growth mechanism of spontaneous vertical growth of ReS_2_ nanosheets from theoretical and experimental insight (Figure 7e).^[^ ^127^ ^]^ It was found that the governing mechanism for the vertical growth of ReS_2_ involves two distinct stages. In the first stage, ReS_2_ grows parallel to the growth substrate. However, the subsequent vertical growth is nucleated at points on the lattice where Re atoms are "pinched" together. At such sites, an additional Re atom binds with the cluster of pinched Re atoms, leaving an under‐coordinated S atom protruding out of the ReS_2_ plane. This under‐coordinated S is “reactive” and binds to free Re and S atoms, initiating growth in a direction perpendicular to the ReS_2_ surface. Using DFT and binding energy calculations, the author explained the anisotropy in the out‐of‐plane growth wherein maximal vertical growth occurs at the center of the predeposited flat ReS_2_ flake and is almost nonexistent along the flake edges. The growth model developed in this work could apply to a broad class of material systems with distorted 1T phase, wherein an out‐of‐plane metal‐metal bond causes an under‐coordinated chalcogen atom to be displaced, triggering vertical growth from the surface of the flat flake. Above research works presented a clear understanding about the out‐of‐plane growth behavior of ReX_2_, which is of significant importance to tailored design of vertical ReX_2_ nanoarrays for diverse functional device applications.

The vertical 3D ReX_2_ arrays with high surface‐to‐volume ratio and edge exposure make them ideal candidates in electrocatalysis and battery applications as discussed in section 4.2. Moreover, the vertical ReS_2_ arrays also show great application potential in sensor, field emission, photothermal radiotherapy and solar‐based disinfection of bacteria, etc.^[^ ^28^, ^126^, ^128^ ^]^ Together with the advantages of spontaneous out‐of‐plane growth of ReX_2_ and the developments of large‐scale fabrication techniques, these materials will exhibit more abundant applications.

### 3.6 ReX_2_‐Based 2D Alloys

Realizing the controllable modulation of energy band structure and electrical conduction is of crucial importance to tailored design of 2D materials for diverse device applications. Alloying of multi‐component metals or chalcogen elements with different stoichiometries in 2D materials provides a versatile and efficient approach for continuously tuning the bandgaps in a wide energy range. As such, researchers have successfully synthesized a series of 2D TMDs alloys (such as MoS_2_ *_x_*Se_2(1‐_ *_x_*_)_, WS_2_ *_x_*Se_2(1‐_ *_x_*_)_, Mo*_x_*W_1‐_ *_x_*S_2_, and Mo_1−_ *_x_*W*_x_*Se_2(1−y_)S_2y_, etc.) which exhibit great feasibility in the tunability of energy band structure, carrier concentration and conduction type.^[^ ^129^, ^130^, ^131^ ^]^ Notably, current research works are mainly focused on the 2H phase TMDs alloys. In this regard, 1T′ phase ReX_2_ which shows distinctive phase structures and properties compared with 2H TMDs provides new opportunities to enrich the structures and properties of 2D TMDs alloys.

**Figure 8:**
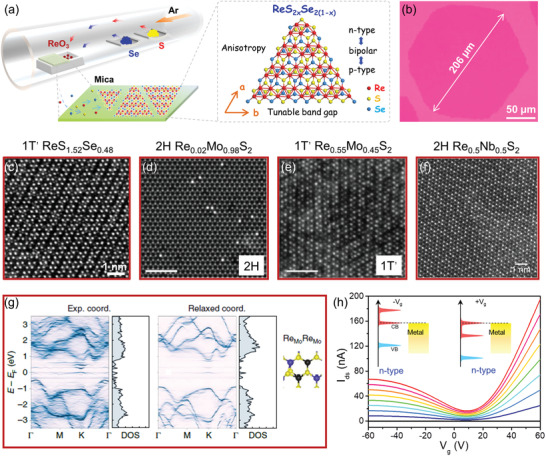
a) Schematic illustration of the atomic structure of ReS_2_ *_x_*Se_2(1−_ *_x_*_)_ alloy and its CVD synthesis approach. b) Typical optical image of a ReS_2_ *_x_*Se_2(1−_ *_x_*_)_ grain. Reproduced with permission.^[^ ^132^ ^]^ Copyright 2016, Wiley‐VCH. STEM images of c) 1T′ ReS_2_ *_x_*Se_2(1−_ *_x_*_)_ alloy, d) 2H Re_0.02_Mo_0.98_S_2_ alloy, e) 1T′ Re_0.55_Mo_0.45_S_2_ alloy, and f) 2H Re_0.5_Nb_0.5_S_2_ alloy. Reproduced with permission.^[^ ^132^, ^136^, ^140^ ^]^ Copyright 2017, Wiley‐VCH. Copyright 2018, Wiley‐VCH. Copyright 2019, American Physical Society. g) Electronic band structures of MoS_2_ containing two Re dopants calculated from experimental 3D atomic coordinates of 6 × 6 × 1 unit cells. Reproduced with permission.^[^ ^138^ ^]^ Copyright 2020, Springer Nature. h) Transfer curves (*I*_ds_–*V*_g_) of Mo_0.98_Re_0.02_S_2_ alloy FET. The *V*_ds_ in changes from 0 to 2 V with an interval of 0.25 V. Reproduced with permission.^[^ ^137^ ^]^ Copyright 2020, Wiley‐VCH.

In view of ReS_2_ and ReSe_2_ have disparate bandgap and opposite carrier type (ReS_2_: 1.6 eV, n‐type; ReSe_2_: 1.3 eV, p‐type), that offer a reasonable route to modulate the bandgap and conduction type by alloying of them. Our group successfully synthesized the spatially composition‐modulated monolayer ReS_2_ *_x_*Se_2(1−_ *_x_*_)_ alloys by systematically adjusting the mass ratio of S and Se powders during the CVD growth process (**Figure** **8**a).^[^ ^132^ ^]^ The size of alloy domain can up to a few hundred microns (≈206 µm) as shown in Figure 8b. The STEM image of ReS_1.52_Se_0.48_ alloy shows that the 1T′ structure is well maintained and the diamond shaped Re4 chains can be visualized clearly (Figure 8c). The bandgaps of ReS_2_ *_x_*Se_2(1−_ *_x_*_)_ alloys were continuously modulated from 1.32 to 1.62 eV along with the composition *x* varying from 0 to 1. Especially, the carrier type, threshold voltage and carrier mobility of ReS_2_ *_x_*Se_2(1‐_ *_x_*_)_ alloy FETs can be systematically modulated by tuning the alloy composition. Furthermore, using the synergy of salt assistance with the confined space CVD strategy, high‐quality and large‐size ReS_2_ *_x_*Se_2(1−_ *_x_*_)_ monolayer crystals can be directly grown on SiO_2_/Si substrates.^[^ ^133^ ^]^ Owing to the low‐symmetry 1T′ structure and tunable bandgap of ReS_2(1−_ *_x_*_)_Se_2_ *_x_* alloys, they exhibit strong anisotropic optical, electrical, and photoelectric properties,^[^ ^132^, ^134^ ^]^ as well as broadband photoresponse to visible and near‐infrared light.^[^ ^133^ ^]^

Beside 1T′ ReS_2_ *_x_*Se_2(1‐_ *_x_*_)_ alloys, constructing 2D alloys from two parent TMDs materials with different phase structures have attracted great attention, as the phase structure can provide more degree of freedom for engineering their structures and properties.^[^ ^135^ ^]^ Ajayan and coworkers demonstrated the successful synthesis of 2D Mo_1−_ *_x_*Re*_x_*Se_2_ and Re*_x_*Mo_1‐_ *_x_*S_2_ alloys with different compositions by alloying 2H phase MoSe_2_/MoS_2_ and 1T′ phase ReSe_2_/ReS_2_ via the CVD technique.^[^ ^29^, ^136^ ^]^ Unlike the widely studied 2H phase TMDs alloys, the Mo_1−_ *_x_*Re*_x_*Se_2_ and Re*_x_*Mo_1‐_ *_x_*S_2_ alloys showed a structural phase transformation from 2H to 1T′ phase as a function of Re doping. Notably, the Re content in the 1T′ structures was found to be controllable between 55% and 100%, while only a small amount of Re (2–4%) was incorporated in the 2H phase. According to the energy calculations and the crystal field theory, a transition from 2H to 1T′ phase is predicted to be more energetically favorable when the Re/(Re+Mo) concentration is greater than ≈50%, which is consistent with the above experiment observations.^[^ ^29^, ^136^ ^]^ Figure 8d,e shows the STEM images of two representative alloys of 2H Re_0.02_Mo_0.98_S_2_ and 1T′ Re_0.55_Mo_0.45_S_2_, respectively. As emphasized in the above work, the stable 1T′ phase MoS_2_ which is metastable in nature can be obtained by alloying with Re when Re‐content reached the ≈50% level. As a result, 1T′ Re_0.55_Mo_0.45_S_2_ alloy shows enhanced HER activity on the basal plane with a low onset potential and a small Tafel slope as well as excellent stability, which is even superior to the benchmark lithium intercalated 1T phase MoS_2_. This study provides a new strategy to improve the overall HER performance of MoS_2_‐based materials via alloying/doping. Furthermore, through precisely tuning the growth thermodynamics of Mo*_x_*Re_1‐_ *_x_*S_2_ alloys, two serious problems, the phase separation and structure reconstruction were successfully suppressed, which are usually neglected but critical to exploring their intrinsic properties.^[^ ^137^ ^]^

The tunable phase structures and the distinct valence electron structures of Mo/W and Re endow 2D ReX_2_‐based alloys many attracting electronic band structure features, photonic and electronic properties.^[^ ^137^, ^138^, ^139^, ^140^, ^141^, ^142^ ^]^ Figure 8g presents the electronic band structures calculated from experimental 3D atomic coordinates including dopant‐free MoS_2_ structure, single Re dopant, two Re dopants and single Re dopant with a S vacancy.^[^ ^138^ ^]^ The band structures show highly distorted indirect bandgaps or metal‐like behavior with a large number of shadow bands. Moreover, based on the high‐quality Mo*_x_*Re_1‐_ *_x_*S_2_ alloys, several novel energy band structure features were revealed, such as subgap formation, Burstein‐Moss effect, especially strong band bowing effect from 1T′ to 2H phase transition.^[^ ^137^ ^]^ What's more, the Mo*_x_*Re_1‐_ *_x_*S_2_ alloys FET devices revealed tunable conduction transition from n‐type to bipolar and p‐type in 1T′ phase, as well as novel "bipolar‐like" electron conduction behavior in 2H phase (Figure 8h). Furthermore, using salt‐assisted CVD growth, Zhou and coworkers synthesized Re*_x_*W_(1−_ *_x_*_)_S_2_ alloys with different compositions which show greatly enhanced responsivity than that of CVD‐grown WS_2_.^[^ ^139^ ^]^ Zettl and coworkers reported the composition‐dependent bandgap of monolayer Re*_x_*Nb_1−_ *_x_*S_2_ in the 2H and 1T′ phases. It shows a broad range of bandgaps, from metallic (*x* = 0, NbS_2_) to semiconducting (*x* = 1, ReS_2_), in contrast to a limited bandgap range offered by isovalent TMDs alloys.^[^ ^140^ ^]^ The above research works highlight the unique alloying effects which do not exist in the single‐phase 2D alloys, and provide the feasibility for potential applications in building novel electronic and optoelectronic devices.

For 2D alloy materials, quantify the spatial and local distribution of atoms is much critical to investigate their structure and properties at the atomic scale. Since the low‐symmetry of 1T′ ReX_2_, the local coordination and elemental distribution in ReX_2_‐based alloys would be significant difference to that of high‐symmetry 2H TMDs alloys. As expected, our group discovered an interesting "sub‐nanometer‐scale local atomic distribution" in the CVD‐grown ReS_2_ *_x_*Se_2(1‐_ *_x_*_)_ alloys. It was found that the Se atoms are preferentially located at the outside of Re4‐chain, while the S atoms are preferentially located at the inside of Re4‐chain. The underlying reason for the preferential S/Se occupation in 1T′ ReS_2(1−_ *_x_*_)_Se_2_ *_x_* alloys is attributed to the stability of different Se/S coordination configurations, where the energy for Se atoms substitution of S atoms is much lower at the relatively larger volume sites.^[^ ^143^ ^]^ Furthermore, an interesting frustration and atomic ordering feature were discovered in mechanically exfoliated monolayer Re*_x_*Nb_(1−_ *_x_*_)_S_2_ alloys.^[^ ^140^ ^]^ Interestingly, the induced atomic ordering can be used as another degree of freedom to considerably modify the bandgap of monolayer semiconductor alloys. Therefore, the local coordination and elemental distribution features of above ReX_2_‐based alloys would offer many novel properties and applications, which are worth to further explore in the future.

### 3.7 ReX_2_‐Based Heterostructures

Various 2D materials can be flexibly combined to build diverse heterostructures with tunable band alignment, opening up giant opportunities for exploring novel properties as well as potential applications in optoelectronics.^[^ ^144^, ^145^ ^]^ In the past several years, vast permutations of heterostructures based on ReX_2_ have been created with novel electronic and optoelectronic properties and applied in photovoltaic cells, rectifiers, photodetectors, light‐emitting diodes, and even catalysis, etc.^[^ ^146^, ^147^, ^148^, ^149^, ^150^, ^151^ ^]^ According to the structure geometries, ReX_2_‐based heterostructures can be divided into two categories: vertical heterostructure and lateral heterostructure. The vertical heterostructure which is bonded by weak Van der Waals interaction can be easily fabricated by assembling ReX_2_ with other 2D materials via mechanical exfoliation and restacking procedure. This flexible approach is suitable for studying the fundamental properties of heterostructures, but difficult to realize the large‐scale device applications, and it is unable to create lateral heterostructures. Besides, CVD growth which is considered as an effective way to realize large‐scale preparation of 2D materials, has been widely used to fabricate both vertical and lateral ReX_2_‐based heterostructures with clean and atomically sharp interface. In this section, we will mainly introduce the research progress of ReX_2_‐based heterojunctions prepared by CVD growth and briefly discuss their electronic and optoelectronic properties.

**Figure 9:**
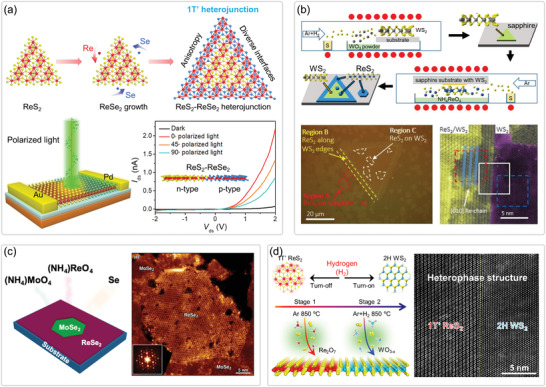
a) Schematic illustration for the synthesis of 1T′ ReS_2_‐ReSe_2_ heterostructure through two‐step epitaxial growth, and the corresponding optoelectronic properties. Reproduced with permission.^[^ ^152^ ^]^ Copyright 2018, Wiley‐VCH. b) Schematic diagram of the two‐step CVD growth of 1T′‐2H ReS_2_‐WS_2_ heterostructures, and the corresponding STEM image. Reproduced with permission.^[^ ^30^ ^]^ Copyright 2017, Wiley‐VCH. c) Schematic diagram for synthesizing MoSe_2_‐ReSe_2_ heterojunction via a one‐pot growth process, and the corresponding colored STEM image. Reproduced with permission.^[^ ^153^ ^]^ Copyright 2019, American Chemical Society. d) Schematic illustration for synthesizing 2H‐1T′ WS_2_‐ReS_2_ heterostructures via a hydrogen‐triggered one‐pot growth process, and the corresponding STEM image. Reproduced with permission.^[^ ^154^ ^]^ Copyright 2020, Wiley‐VCH.

The unusual 1T′ phase structure of ReX_2_ offers new possibilities to construct both the homophase (1T′–1T′) and heterophase (1T′–2H) heterostructures for exploring novel interface structures and electrical properties. As shown in **Figure** **9**a, our group reported the synthesis of 1T′ ReS_2_‐ReSe_2_ lateral heterostructure by two‐step CVD growth on mica substrate.^[^ ^152^ ^]^ The obtained heterojunction shows high crystal quality with an atomically sharp interface. Interestingly, three types of epitaxy growth modes accompanying formation of three different interfaces were revealed in the growth of 1T′ lateral heterostructures, where the angles between the *b*‐axis of ReS_2_ and ReSe_2_ are 0°, 120°, and 180°, respectively. Electrical transport demonstrates that the 1T′ ReS_2_‐ReSe_2_ heterostructure forms lateral p‐n junction with intrinsic rectification characteristics and exhibits polarization‐dependent photodiode properties.

The stable 1T′ phase structure of ReX_2_ provides great opportunities for building 2D heterophase structures with other 2H phase TMDs, which could bring abundant interface structures and novel properties. Tongay and coworkers reported the first synthesis of ReS_2_‐WS_2_ pseudo‐1D‐2D lateral heterophase structures via a two‐step CVD process on sapphire substrate (Figure 9b).^[^ ^30^ ^]^ Structure characterization of the interface between these dissimilar 2D materials reveals that ReS_2_ lateral heterojunctions to WS_2_ produce well‐oriented (highly anisotropic) Re‐chains perpendicular to WS_2_ edges. Notably, the lateral heterostructure conforms around the entire WS_2_ edges followed by the formation a few of vertical heterostructures. Moreover, serious surface‐deposition exists at the defect‐rich 1D interface of the heterostructure where exists high‐density nucleation centers. As is well known, the surface‐deposition is an inevitable problem in the synthesis of 2D heterojunction via two‐step CVD growth, and such problem is further aggravated in the synthesis of heterophase junction because of the large lattice mismatch. Ajayan and coworkers addressed the synthesis of 1T′‐2H ReSe_2_‐MoSe_2_ lateral heterophase structure on sapphire substrate by one‐step CVD growth with NH_4_ReO_4_ and (NH_4_)_2_MoO_4_ as Re and Mo precursors (Figure 9c).^[^ ^153^ ^]^ The authors utilized the phase diagrams of the binary Mo‐Re system to differentiate it from commonly reported Mo‐W chalcogenide systems toward its unique thermodynamically dictated preference for alloy versus heterostructure formation at the monolayer limit. Though atomic sharp heterophase interface can be obtained, interdiffusion of Re and Mo atoms in the two phases still occur, and plenty of defects exist in the entire heterojunctions. Actually, the defect and alloying problems are intrinsic drawbacks of heterostructures prepared via one‐step growth. Recently, our group developed a hydrogen‐triggered one‐pot growth approach to synthesize 2H‐1T′ WS_2_‐ReS_2_ heterophase junctions with high‐quality interface structure (Figure 9d).^[^ ^154^ ^]^ The sequential introduction of hydrogen during growth system, which acts as a “switch” to selectively turn off the growth of ReS_2_ while turning on the growth of WS_2_, allows WS_2_ to seamlessly grow around ReS_2_ to form the lateral WS_2_‐ReS_2_ heterojunction. As a result, an atomically sharp 2H‐1T′ heterophase interface was obtained and no cross‐doping existed in the two phase regions as confirmed by the Raman, PL and STEM characterization. Moreover, the outer WS_2_ grains are preferred to nucleate at the vertices of inner ReS_2_ grain with fixed lattice orientation, promoting the merger of surrounding WS_2_ grains to form a single crystal. Based on the high‐quality WS_2_‐ReS_2_ heterophase junction, effective photocarriers separation, prominent rectification characteristics, and polarization‐sensitive photodiode properties were achieved.

Beside above lateral heterojunctions, several research works also reported the synthesis of 2D ReX_2_‐based vertical heterojunctions. Fu and coworkers reported the synthesis of ReS_2_‐WS_2_ vertical heterostructures using an innovative twinned growth relationship between ReS_2_ and WS_2_.^[^ ^155^ ^]^ For the twinned growth of ReS_2_‐WS_2_ vertical heterostructures, Au was chose as the growth substrate and W‐Re alloy foil was used as the Re and W sources, which lower the barrier energies for this special twinned growth process. With this approach, 100% overlap for each of the stacked ReS_2_‐WS_2_ heterostructures with crystal size up to 600 µm^2^ were achieved. Interestingly, owing to the weak interlayer van der Waals interactions, the ReS_2_‐WS_2_ vertical heterostructures can be disassembled into individual building blocks under the assistance of disassembly promoters (Te).^[^ ^156^ ^]^ Moreover, Ye and coworkers reported the growth of Se nanoplates on monolayer ReS_2_ through a two‐step CVD process to build the Se‐ReS_2_ vertical heterostructures.^[^ ^157^ ^]^ The Se nanoplates could conjugate with underlying ReS_2_ via strong chemical hybridization at heterointerface due to the existed defects in ReS_2_, which allow it grown with the [001] axis vertically aligned with underlying ReS_2_. In addition, utilizing the advantage of atomically smooth and chemically inert surface of graphene, graphene‐ReS_2_ vertical heterostructures were achieved via CVD growth.^[^ ^158^ ^]^ Compared with rough surface of SiO_2_/Si substrate with dangling bonds which hinders the uniform growth of ReS_2_, the inert and smooth surface of graphene provides lower energy barrier for migration of the adatoms, thereby promoting the in‐plane growth of ReS_2_ on it. In‐situ patterning of the graphene‐ReS_2_ heterostructure was achieved by the selective growth of ReS_2_ on the patterned graphene, which is attributed to the strong binding energy between sulfur atoms and graphene surface.

Overall, the above research works provide useful insights for understanding the growth dynamics and stability of ReX_2_‐based heterostructures from chemically, structurally, and electronically different phases. Present studies are mainly focused on developing different synthesis strategies to build novel 2D heterostructures with diverse interface structures, while exploring their novel electronic and optoelectronic properties are still quite scarce. Moreover, the diverse phase structures and abundant properties of group‐VI TMDs and ReX_2_ offer significant opportunities to build a new class of 2D heterophase structures for engineering their interface structures and energy band alignments. In this regard, further exploring novel interface structures, properties and applications of 2D ReX_2_‐based heterostructures should be the main emphasis of future research works. Furthermore, the synthesis approach for obtaining large‐scale and highly uniform 2D ReX_2_‐based heterostructures with desired interface structures (lateral or vertical heterojunction) still needs to be optimized. It could be optimistically anticipated that various epitaxial growth methods as well as superior properties of abundant ReX_2_‐based heterostructures would spring out in the near future.

## 4 Application of ReX_2_

This section is devoted to specifically discuss the various applications of ReX_2_. For this purpose, we categorized all reported applications of ReX_2_ into three main areas based on the structural and functional features of each application. The pertinent subsections including electronic and optoelectronic devices, energy conversion and storage, as well as other emerging applications.

### 4.1 Electronic and Optoelectronic Devices

2D ReX_2_ materials profit from their unusual 1T′ structure, finite bandgap, novel optical and electrical properties have attracted considerable research interest in constructing next‐generation solid‐state electronic devices.^[^ ^13^, ^19^ ^]^ In this subsection, we focus on three primary applications of ReX_2_ in the field of electronic and optoelectronic devices: field effect transistors (FETs), logic circuits, and photodetectors.

#### 4.1.1 Field Effect Transistors

**Figure 10:**
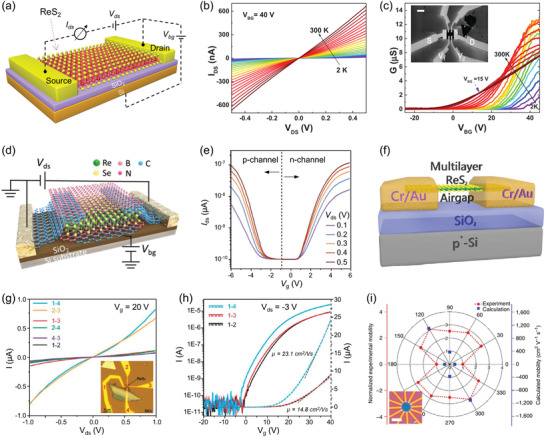
a) Schematic structure of ReX_2_ FET. b) Double sweeps of *I*_DS_–*V*_DS_ characteristics for several temperatures at fixed *V*_BG_ = 40 V with negligible hysteresis. c) Temperature dependence of the sheet conductivity on back‐gate voltage. Inset shows the SEM image of the FET device. Reproduced with permission.^[^ ^21^ ^]^ Copyright 2015, Wiley‐VCH. d) Schematic diagram of a ReSe_2_ FET encapsulated with hBN. e) Transfer characteristics for ReSe_2_ FET at different *V*_bg_. Reproduced with permission.^[^ ^160^ ^]^ Copyright 2019, Wiley‐VCH. f) Schematic of suspended ReS_2_ device. Reproduced with permission.^[^ ^161^ ^]^ Copyright 2018, American Chemical Society. The direction‐dependent g) output curves and h) transfer curves of ReS_2_ device. Reproduced with permission.^[^ ^15^ ^]^ Copyright 2015, American Chemical Society. i) Normalized field‐effect mobility of a six‐layer device along 12 directions evenly spaced at 30° apart plotted in polar coordinates (red dots with left axis). The direction with the lowest mobility was set to be the 0° (or 180°) reference. The optical image of the device is shown in the inset. Reproduced with permission.^[^ ^19^ ^]^ Copyright 2015, Springer Nature.

FETs play an important role in our modern information technology as one of the most effective technologies of all time and have facilitated the development of computers, the Internet, thin mobile displays, and much more.^[^ ^159^ ^]^ Atomically thin 2D TMDs as channel materials in FETs can eliminate drain‐induced short‐channel effects to achieve high performance. In the past several years, FETs based on mechanically exfoliated ReS_2_ have been reported with a current on/off ratio up to 10^7^ and electron mobility varying from 0.1 to 18 cm^2^ V^−1^ s^−1^, the device is schematically shown in **Figure** **10**a. The *I*_DS_ varies linearly with *V*_DS_ in the output curves measured at different temperatures (Figure 10b), indicating well‐developed contact between the electrodes and the ReS_2_ channel. The dependence of the channel conductance G on *V*_BG_ at different temperatures in Figure 10c shows an interesting metallic state associated with the metal‐to‐insulator transition due to the increase of Fermi level when *V*_BG_ is larger than 15 V. Moreover, FET devices based on the CVD‐grown ReS_2_ have also been extensively investigated, the mobility varies from 0.072 to 40 cm^2^ V^−1^ s^−1^ depending on the crystal quality of the samples prepared in different works. Moreover, FETs based on ReSe_2_ exhibit a p‐type conduction behavior with mobility in the range of 1.36 × 10^−3^–14.1 cm^2^ V^−1^ s^−1^ which is relatively lower than that of ReS_2_ and most 2D TMDs. The relatively lower carrier mobility of ReX_2_ attributed to its large electron effective mass may restrict its high‐performance electronic device applications. Certainly, the carrier mobility of ReX_2_ can be further improved by the chemical modification of materials or the optimization of the electrode‐material contact. Anyway, the different bandgap together with the opposite conductive features of ReS_2_ and ReSe_2_ provides great perspectives for optoelectronic applications, although their mobility still has great room for improvement.

Several strategies have been adopted to optimize the performance of ReX_2_‐based FET devices. Using graphene as contact electrodes, ambipolar behavior of ReSe_2_ transistors with electron and hole mobilities of 1.1 and 3.1 cm^2^ V^−1^ s^−1^, respectively, can be achieved due to the tunable Fermi level of graphene by electrical field.^[^ ^160^ ^]^ Xiu and coworkers constructed a top‐gated ReSe_2_ FET device with high‐*κ* Al_2_O_3_ as dielectric layer, which achieved a significant improvement of the electron mobility over 500 times and the hole mobility over 100 times at low temperature.^[^ ^23^ ^]^ Moreover, by fabricating ReS_2_ FET device onto h‐BN substrate, the on/off switching ratio can reach to 10^8^ and the mobility up to 30 cm^2^ V^−1^ s^−1^.^[^ ^22^ ^]^ It is the reduced charged impurities that decrease the charge scattering of ReS_2_ on h‐BN substrate and thus accounting for the enhancement of mobility. The h‐BN encapsulated ReSe_2_ FETs, which isolate the ReSe_2_ channel from both SiO_2_/Si substrate and air, can ulteriorly improve the device performance (Figure 10d,e).^[^ ^160^ ^]^ Constructing suspended ReS_2_ FET device is also an effective method to eliminate the interface traps between ReS_2_ and SiO_2_/Si substrate for obtaining more intrinsic electrical properties of ReS_2_ (Figure 10f).^[^ ^161^ ^]^ Liu and coworkers reported the monolayer ReS_2_ FET mobility up to 40 cm^2^ V^−1^ S^−1^ via measuring at low temperature condition which can reduce the electron‐phonon scattering.^[^ ^20^ ^]^ Furthermore, Park and coworkers achieved a better‐performance ReS_2_ FET by simple oxygen plasma treatment.^[^ ^84^ ^]^ The current on/off ratio enhancing from 10^3^ to 10^4^ and mobility enhancing from 4 to 7.6 cm^2^ V^−1^ s^−1^ via increasing the O_2_ plasma treatment time from 0 to 60 s. The improvements are attributed to the surface oxidation induced electron‐accepting sites, and consequently cause the p‐type doping of ReS_2_, which reduced the electron injection from metal to ReS_2_.

Different from conventional isotropic 2D materials like MoS_2_, the effective mass of electron and hole of ReX_2_ varies along different crystal directions owing to the unusual low‐symmetry crystal structure.^[^ ^15^, ^120^, ^162^ ^]^ It affords ReX_2_ inspiring 2D in‐plane anisotropic electronic properties which have been revealed by fabricating ReX_2_ FETs with multipair probes configuration and performing angle‐resolved transport measurements. For example, Suenaga and coworkers investigated the relationship between crystalline orientation of ReS_2_ and the electron transport by combination of transport measurement and transmission electron microscopy (Figure 10g,h).^[^ ^15^ ^]^ It is found that the conductance and electron mobility were strongly dependent on the crystalline orientation. The conductance along the *b*‐axis direction (0.82 *μ*S) was an order of magnitude higher than that parallel to the a‐axis (0.075 *μ*S). The electron mobility along the *b*‐axis (23.1 cm^2^ V^−1^ s^−1^) was nearly two times higher than that crossing the *b*‐axis (14.8 cm^2^ V^−1^ s^−1^). In the following study, the anisotropic ratio of polarization mobility at different crystal orientations reached up to ≈3 which is the highest value compared with other anisotropic 2D materials (Figure 10i).^[^ ^19^ ^]^ Besides ReS_2_ material, the ReSe_2_ and even the Re‐based 2D TMDs alloys and heterostructures with 1T′ structure also show anisotropic electrical transport properties.^[^ ^132^, ^152^ ^]^ It is worth noting that the strong anisotropic electronic characteristics of ReX_2_ open up new opportunities for constructing novel FETs and logical devices.

#### 4.1.2 Logical Circuits

**Figure 11:**
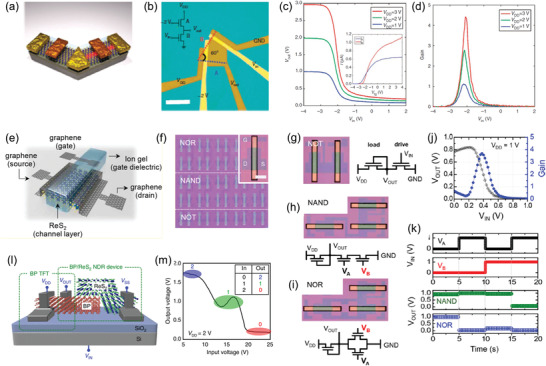
a) Schematic diagram of the structure of an inverter combining two top‐gated anisotropic ReS_2_ FETs. b) Optical image of a typical inverter device, scale bar 10 µm. Inset: the circuit diagram of the inverter. Inset: the circuit diagram of the inverter. c) Transfer characteristics of an inverter operated at *V*_DD_ = 1, 2, and 3 V. Inset: The transfer curves of two FETs with 100 mV of *V*_ds_, confirming the anisotropic behavior. d) The signal gain of the inverter extracted from c. Reproduced with permission.^[^ ^19^ ^]^ Copyright 2015, Springer Nature. e) Schematic diagram of the CVD‐grown ReS_2_ transistors with graphene electrodes and ion gel gate dielectrics. f) Optical top‐view image of the ReS_2_ transistors and logic gates, such as NOR, NAND, and NOT gates. The inset presents an optical image of a single ReS_2_ transistor with graphene drain, source, and gate electrodes and an ion gel gate dielectric. g–i) Optical top‐view image and schematic band diagram of the g) NOT, h) NAND, and i) NOR gates gate. j) Voltage transfer characteristics of the NOT gate and signal gain at *V*_D_ = 1 V. k) The input and output voltages of the NAND and NOR gates as a function of time. Reproduced with permission.^[^ ^164^ ^]^ Copyright 2017, American Chemical Society. l) Schematic illustration of the phosphorene/ReS_2_ ternary inverter. m) *V*_IN_ versus *V*_OUT_ characteristic of the ternary inverter. The inset shows an input–output table of the ternary inverter. Reproduced with permission.^[^ ^146^ ^]^ Copyright 2016, Springer Nature.

These preliminary works mentioned in the previous subsection have aroused great interest in the applications of 2D ReX_2_ in smart integrated circuits toward practical microprocessors. The key step to this application is the development of basic logic gates, especially the inverter (NOT gate). For the anisotropic ReX_2_, the lattice orientation could be used as an alternate design variable to turn transport properties and optimize performance of 2D integrated circuits. Xing and coworkers fabricated the first ReS_2_‐based digital inverter which is composed of two anisotropic ReS_2_ FETs along the a‐axis and *b*‐axis of a quadrilateral‐shaped ReS_2_ flake with a 60° inner angle as shown in **Figure** **11**a.^[^ ^19^ ^]^ Figure 11b presents the OM image of the inverter device, where HfO_2_ and Au were used as insulating dielectric layer and top gate electrode, respectively. The voltage transfer curves (Figure 11c) show excellent logic‐level conservation between logic 0 and 1 at different input voltages (*V*_in_). The signal gain (Figure 11d) of the inverter reaches 4.4 when the supply voltage (*V*_DD_) is 3V, indicating its outstanding sensitivity to the changes of *V*_in_. This work provided a way to regulate the performance of logic circuits based on different crystal orientations of low‐symmetry ReS_2_ material, and injected fresh blood into the research field of 2D ReX_2_ materials. In general, a Schottky barrier exists at the interface between ReS_2_ material and metal, which degrades the efficiency of carriers injection from channel to electrode. Quantum transport simulation shows that selecting appropriate electrode materials which have strong interface interaction and relatively smaller work function for ReS_2_ with an Ohmic contact could improve the FET performance for logic circuits application.^[^ ^163^ ^]^ In experiment, Cho and coworkers adopted CVD‐grown graphene as the electrodes to fabricate large‐scale low‐voltage‐operated ReS_2_ transistors and logic gates based on CVD‐grown large‐area multilayer ReS_2_ film (Figure 11e,f).^[^ ^164^ ^]^ The contact resistance of the ReS_2_ transistors with graphene electrodes decreased dramatically compared with that prepared with metal Cr electrodes. Then, the multiple ReS_2_ transistors were successfully assembled to build the logic devices, including NOT, NAND, and NOR gates (Figure 11g–i). Notably, the signal inverter gain of NOT logic gate exceeded 3.5 (Figure 11j), which is sufficient to drive a next‐stage component in a logic circuit. For NAND and NOR logic gates, the input and output voltages as a function of time exhibit excellent switching capacities between logic state "0" and logic state "1" (Figure 11k), indicating the outstanding computing capacities. Park et al. demonstrated a ternary inverter as multi‐valued logic application by constructing a negative differential resistance device based on phosphorene/ReS_2_ heterojunction (Figure 11l).^[^ ^164^ ^]^ Three distinct logic values (states "2," "1" and "0") were achieved in the *V*_IN_ versus *V*_OUT_ characteristic of the device (Figure 11m). The above research progresses demonstrate that it is possible to construct a wide variety of complex functional digital logical circuits using ReX_2_ materials in the future.

#### 4.1.3 Photodetectors

Photodetector, a key component in many devices, has a wide range of applications in various fields including military and national economy. The working principle of most photodetectors is based on photoelectric effect, which can convert the information stored in light into electrical signals that can be processed by standard electronic devices.^[^ ^165^ ^]^ For photodetector based on 2D materials, the incident photons with energy larger than its bandgap can excite bound excitons which are separated by built‐in field or applied electrical field and ultimately generate photocurrent.^[^ ^166^ ^]^ The large bandgap (ReS_2_: ≈ 1.3 eV and ReSe_2_: ≈ 1.6 eV) and the superior conductivity of ReX_2_ make them ideal candidates for constructing high‐performance photodetectors. Furthermore, the anisotropic structure of ReX_2_ can afford the polarization state into its photodetection, and thus increase the detection dimension to get more accurate detection information. In this regard, ReX_2_ material would have broad application prospects in the field of photodetection.

**Figure 12:**
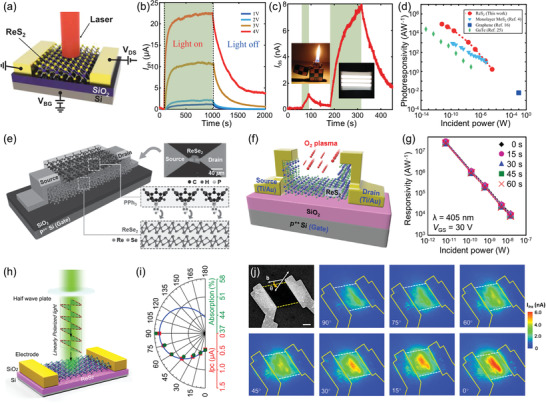
a) Schematic structure of ReS_2_ back‐gate photodetector. Reproduced with permission.^[^ ^21^ ^]^ Copyright 2015, Wiley‐VCH. b) Photocurrent response of the ReS_2_ photodetector under various *V*_ds_, fixing *P*_light_ at 20 nW and *V*_bg_ at 50 V. c) Weak signal detection in the ReS_2_ photodetector. d) The dependence of *R_P_* on *P*_light_. For comparison, the results from monolayer MoS_2_, multilayer GaTe, and graphene are also presented. Reproduced with permission.^[^ ^22^ ^]^ Copyright 2015, Wiley‐VCH. e) Schematic diagram and optical image of back‐gated ReSe_2_ devices doped by PPh_3_ and a descriptive illustration for the n‐doping mechanism by PPh_3_ at the PPh_3_/ReSe_2_ interface. Reproduced with permission.^[^ ^168^ ^]^ Copyright 2016, Wiley‐VCH. f) Schematic illustration of O_2_‐plasma‐treated ReS_2_ FET. g) Photoresponsivity of the ReS_2_ photodetectors as a function of incident laser power according to various O_2_ plasma treatment times. Reproduced with permission.^[^ ^84^ ^]^ Copyright 2015, Wiley‐VCH. h) 3D schematic view of the photodetection device. i) The photocurrent with drain bias of 1 V (red squire) and absorption (green circle) measured under different polarization angle of green light and plotted in polar coordinates. The blue lines are the fitting results using sinusoidal function. Reproduced with permission.^[^ ^20^ ^]^ Copyright 2015, Wiley‐VCH. j) SEM image of the ReSe_2_ photodetector followed by polarization‐dependent photocurrent mapping of the device, showing prominent linear dichroic photodetection. Reproduced with permission.^[^ ^112^ ^]^ Copyright 2016, American Chemical Society.

In the past several years, plenty of ReX_2_‐based photodetectors with diverse structures have been fabricated to explore their practical applications. **Figure** **12**a shows the schematic structure of ReX_2_ photodetector which is a standard photoconductor‐type detector.^[^ ^21^ ^]^ Here, we focus on the three parameters: responsivity (*R*), external quantum efficiency (EQE) and response time (*τ*: rising (*τ*_r_) and decaying (*τ*_d_)), which are the primary indicators for assessing the performance of photodetectors.^[^ ^165^ ^]^ Liu and coworkers reported the first ReS_2_‐based photodetector fabricated by the mechanically exfoliated few‐layer ReS_2_, and the device achieved a responsivity of 1000 A W^−1^.^[^ ^20^ ^]^ Xing and coworkers further presented a high responsivity photodetector based on few‐layer ReS_2_.^[^ ^22^ ^]^ As shown in Figure 12b, the time‐resolved response curve of the ReS_2_ device exhibited a stable and repeatable response to the 532 nm laser illumination but its response time was on the order of seconds. Importantly, the optimized photoresponsivity reached as high as 88 600 A W^−1^ and EQE up to 2 × 10^7^%, which are the record values compared to other individual 2D materials with similar two‐terminal device structures. Taking advantage of the high photoresponsivity of the ReS_2_ photodetectors, it can even be used to detect the weak signals from a lighter and limited fluorescent lighting in a dark room (Figure 12c). Such high photoresponsivity is attributed to the increased light absorption as well as the gain enhancement due to the existence of trap states in the few‐layer ReS_2_ flakes. With the fast development of CVD synthesis of ReX_2_, CVD‐grown ReS_2_ and ReSe_2_ were widely utilized to construct the high‐performance photodetectors. Xiu and coworkers reported the photodetector based on CVD‐grown few‐layer ReS_2_ with the photo responsivity of 16.14 A W^−1^ and the EQE reached 3168% under the irradiation of 633 nm laser at power of 25 nW.^[^ ^21^ ^]^ Notably, the response rate is quite slow, especially the *τ*_d_ is as long as ≈1000 s. Zhai and coworkers explored the light response properties of CVD‐grown single crystal ReS_2_ flake and poly‐crystalline ReS_2_ bilayer film.^[^ ^100^ ^]^ They achieved the responsivity of 604 A W^−1^ and EQE of 1.50 × 10^5^% in the single crystal ReS_2_ flake device, and found that the response rate of the former (*τ*_r_ and *τ*_d_ ≈ 2 ms) is shorter than the latter (*τ*_r_: 5.5 s and *τ*_d_: 11.7 s). In general, photodectors based on mechanical exfoliated and CVD‐grown ReS_2_ routinely achieve high responsivity, indicating high photogain, originating from the long lifetime of photogenerated carriers which are boosted by trap states. In contrast, the photodetectors based on ReSe_2_ have relatively low responsivity (*R*: 2.98–5.1 A W^−1^ and EQE: 4.58 × 10^2^%) due to the low carrier mobility, but exhibit quite fast response rate (both *τ*_r_ and *τ*_d_ at ms level).^[^ ^23^, ^104^ ^]^ Especially, owing to the relatively smaller bandgap (≈1.3 eV) of ReSe_2_, its photodetectors show a broadband photoresponse from visible to NIR (940 nm).^[^ ^101^, ^104^ ^]^ From the above research works, we can see a distinct trade‐off existed between responsivity and response speed of ReS_2_ photodetectors, which is known as the typical feature of photoconductive‐type detector.^[^ ^165^ ^]^ Moreover, the performance of photodetectors based on mechanically exfoliated ReX_2_ is superior to that based on CVD‐grown ReX_2_,^[^ ^22^, ^23^ ^]^ which is reasonable because of the higher crystal quality of exfoliated ReX_2_. The performance of photodetector fabricated by the relatively thicker ReX_2_ is superior to that of monolayer, and the single crystal is better than the poly‐crystalline.^[^ ^100^ ^]^ Despite considerable research works have performed on the optoelectric properties of ReX_2_ material, the relatively low responsivity and slow response time still restrict its usage in high‐sensitive and fast switching applications.

For promoting the applicability of ReX_2_ photodetectors, several recent studies have strived to elevate their performance through modifying the materials or device configuration, such as doping, chemical modification, suspended channel, heterostructure construction, and so on.^[^ ^157^, ^161^, ^167^, ^168^, ^169^, ^170^ ^]^ For instance, a very high performance ReSe_2_ photodetector with a broad detection range (*λ*>1064 nm), high responsivity (1.18 × 10^6^ A W^−1^), and fast temporal response (*τ*_r_: 30 ms and *τ*_d_: 64 ms) was obtained by the modification of (3‐Aminopropyl) Triethoxysilane (APTES) and Triphenylphosphine (PPh_3_), as shown in Figure 12e.^[^ ^168^ ^]^ This high performance is achieved by suppressing the interfacial carrier scattering with APTES treatment and improving the source‐side contact resistance through a PPh_3_‐based n‐doping technique. Similarly, using HCl‐based selective p‐doping technique can also increase the photoresponsivity of ReSe_2_ photodetecor from 79.99 to 1.93 × 10^3^ A W^−1^, and the the *τ*_r_ decreases from 10.5 to 1.4 ms with *τ*_d_ from 291 to 3.1 ms compared with the intrinsic ReSe_2_ device.^[^ ^167^ ^]^ Moreover, Park and coworkers demonstrated a high‐performance ReS_2_‐based photodetector with high photoresponsivity (2.5 × 10^7^ A W^−1^) and fast temporal response (*τ*_r_: 670 ms and *τ*_d_: 5.6 s) through a simple oxygen (O_2_) plasma treatment (Figure 12f,g).^[^ ^84^ ^]^ This improvement originates from the controlled thickness of ReS_2_ and the intentionally created defects (traps) in the ReS_2_ during the O_2_ plasma treatment, which affected the drain current at the off‐state and the recombination of photocarriers. In another work, the molecular O_2_ physisorption can be used as "gating" to modulate the carrier density of single‐layer ReSe_2_ transistors, resulting in a high *R* of 95 A W^−1^ and EQE of 18 645% in O_2_ environment.^[^ ^171^ ^]^ Furthermore, Lodha and coworkers investigated the responsivity and response rate in supported (SiO_2_/Si substrate) and suspended ReS_2_ photodetectors to distinguish the roles of intrinsic bulk traps in ReS_2_ and extrinsic traps at the ReS_2_/SiO_2_ interface.^[^ ^161^ ^]^ It was found that the dominant operating mechanism of these photodetectors can be switched between photogating and photoconductive regimes under gate voltage modulation. As a result, high responsibility of 4 A W^−1^ and much low response time of 20 µs were achieved on the suspended ReS_2_ photodetector.

Beside engineering ReX_2_ itself, the performance of ReX_2_ photodetectors can also be enhanced by combining them with other 0D/1D/2D semiconductors to build heterostructure devices. The responsivity of the ReS_2_ photodetector decorated with 0D CdSe‐CdS‐ZnS quantum dots could be enhanced by more than 25 times (up to ≈654 A W^−1^) and the *τ*_r_ and *τ*_d_ can be also reduced from 12s and 13s to 3.2 s and 2.8 s, respectively.^[^ ^172^ ^]^ The excellent optoelectronic performance is originated from the coupling effect of quantum dots light absorber and cross‐linker ligands 1,2‐ethanedithiol, where the photoexcited electron–hole pairs in quantum dots can separate and transfer efficiently due to the type‐II band alignment. Moreover, the photodetectors based on Se/ReS_2_ heterostructures exhibit ultrahigh detectivity of up to 8 × 10^12^ Jones and fast response time of less than 10 ms, illustrating the great advantage of directly integrated 1D Se based nanostructure on planar ReS_2_ films for optoelectronic applications.^[^ ^157^ ^]^ Moreover, by building a type II WSe_2_‐ReS_2_ p‐n junction, a near‐direct heterointerface bandgap (0.7 eV) was achieved to enhance photogeneration.^[^ ^150^ ^]^ The device exhibits ultrafast response time (5 µs) and high responsivity (3 A W^−1^) at the entire hetero‐overlap region, and broadband photoresponse to IR (1250 nm). **Table** **2** summarizes the main figures‐of‐merit of ReX_2_‐based photodetectors.

**Table 2:** Summary of the performance of ReX_2_‐based photodetectors

	Measurement conditions								
Material	*V*_ds_ [V]	*V*_g_ [V)]	Laser and Intensity	On‐off ratio	Mobility [cm^2^ V^−1^ s^−1^]	Responsivity [A W^−1^]	EQE [%]	Response time	Ref.
FL ReS_2_ on h‐BN^a)^	4.0	−50	532 nm (6 pW)	10^7^	30	88 600	2 × 10^7^	–	^[^ ^22^ ^]^
FL ReS_2_	0.1	30	0.1 mW	10^5^	40	1000	–	–	^[^ ^20^ ^]^
FL ReS_2_ on h‐BN	0.5	10	Deep ultraviolet light	10^7^	45	60	3.4	*τ*_r_ 3.5 s, *τ*_d_ 16 s	^[^ ^169^ ^]^
FL ReS_2_	0.5	10	532 nm (10 nW)	10^4^	20.2	45	10 500	–	^[^ ^170^ ^]^
CVD‐grown FL ReS_2_	0.5	50	500 nm (3.11 mW cm^−2^)	10^2^–10^3^	30	604	1.5 × 10^5^	2 ms	^[^ ^100^ ^]^
CVD‐grown FL ReS_2_	0.1	50	633 nm (25 nW)	10^6^	1	16.14	3168	–	^[^ ^21^ ^]^
CVD‐grown FL ReSe_2_	5	0	808 nm (566 mW cm^−2^)	–	1.36*10^−3^	2.98	458	*τ*_r_ 5.47 s, *τ*_d_ 8.41 s	^[^ ^101^ ^]^
CVD‐grown ML ReSe_2_	1	0	850/940 nm (6.07/6.97 mW cm^−2^)	–	0.98	8.4/5.1	–	*τ*_r_ 23 ms, *τ*_d_ 50 ms	^[^ ^104^ ^]^
Suspend FL ReS_2_	0.5	80	(≈140 mW cm^−2^)	10^4^	≈8	4	–	2 × 10^−5^ s	^[^ ^161^ ^]^
FL ReSe_2_ doped by PPh3	5 V	50	520 nm (5 pW)	–	8.85	3.68 × 10^4^	–	*τ*_r_ 30 ms, *τ*_d_ 64 ms	^[^ ^168^ ^]^
FL ReS_2_ treated by O_2_ plasma	5	30	405 nm (5 pW)	10^4^	11	2.5 × 10^7^	–	*τ*_r_ 0.67 s, *τ*_d_ 5.6 s	^[^ ^84^ ^]^
ML ReSe_2_ adsorbed O_2_	–1	–40	633 nm (100 mW cm^−2^)	–	9.78	95	18 645	*τ*_r_ 68 ms, *τ*_d_ 34 ms	^[^ ^171^ ^]^
CVD‐grown ML ReS_2_ modified by QDs	3	30	589 nm (0.12 mW cm^−2^)	10^5^	9.8	654	–	*τ*_r_ 3.2 s, *τ*_d_ 2.8 s	^[^ ^172^ ^]^

^a)^Acronyms: ML‐monolayer, FL‐few layer, *τ*_r_‐rising time, *τ*_d_‐decaying time.

It has been mentioned that, unlike other isotropic 2D materials, the structure anisotropy induced linear dichroism in ReX_2_ enables researchers to build the polarization‐sensitive photodetectors. Liu et al. demonstrated the first polarization‐dependent photodetector with high photoresponsivity based on atomically thin ReS_2_.^[^ ^20^ ^]^ Figure 12h shows the schematic view of the photodetection device, where the polarization angle of light is controlled by a polarizer and a half‐wave plate. By rotating the polarization of the light while keeping the incident power constant, the photocurrent changes dramatically. The photocurrent with the incident light polarized along the *b*‐axis of ReS_2_ is much stronger than that perpendicular to the *b*‐axis. Notably, the photocurrent and absorption have a quite similar dependence on the incident light polarization, which strongly suggests that the intrinsic polarization‐dependent photoresponse originates from the anisotropic structure of ReS_2_ itself (Figure 12i). Similarly, the polarization‐sensitive photoresponse was also achieved in the few‐layer ReSe_2_ photodetector.^[^ ^23^ ^]^ Figure 12j shows the large‐area photocurrent mapping images for the whole channel under different polarization directions of the incident laser of 633 nm. The photocurrent reached to the maximum (minimum) value when the incident light is polarized parallel (perpendicular) to the *b*‐axis. The ReSe_2_ device also exhibits ambipolar (electron and hole regimes) gate‐tunable linear dichroism photodetection. Moreover, the polarization‐dependent photoresponse was also achieved from ReX_2_‐based alloys and heterostructures.^[^ ^132^, ^152^, ^154^ ^]^ Hence, the robust linear dichroism photodetection with high photoresponsivity reported on the above ReX_2_‐based materials demonstrates a route to exploit the intrinsic anisotropy of 2D materials and opens up new ways for the applications of 2D materials for light polarization detection.

At this stage, a deeper understanding of the photocurrent and carrier recombination mechanisms is needed for the optimization of photodetector performance. From the application perspective, the sizable variation in the figure‐of‐merit reported by different works on the ReX_2_ photodetectors indicates that device fabrication, contact metals and measurement environment play important roles in the photodetection performance. Hence, more sophisticated and fundamental advances for the improvement of device performance of ReX_2_‐based photodetectors are urgently needed.

### 4.2 Energy Conversion and Storage

Developing new energy conversion and storage techniques is highly desirable for addressing the global energy crisis and environmental problems. In order to lessen the reliance on fossil fuels, the production of hydrogen from water splitting has become an important subject as H_2_ has been viewed as the most promising sustainable clean energy source.^[^ ^173^ ^]^ Currently, highly efficient photocatalytic and electrocatalytic hydrogen evolution are considered as the two most important routes to produce H_2_.^[^ ^173^, ^174^ ^]^ Moreover, the new generation batteries have tremendous application prospects in future intelligence power system owing to its high power density and high energy density.^[^ ^175^ ^]^ The rational design of electrode materials is the key to all above devices. Among various electrode materials, 2D layered materials have shown significant advantages in energy conversion and storage applications, i) high specific surface area, ii) abundant active sites, iii) weak interface force between layer and layer, iv) short ion path length, and so on.^[^ ^176^, ^177^, ^178^ ^]^ Compared with most 2H TMDs materials, ReX_2_ materials with stable 1T′ structure and much weaker interlayer coupling make them highly attractive in the applications of energy conversion and storage devices. In this section, we primarily introduce two major application fields of ReX_2_: catalysis and battery.

#### 4.2.1 Photocatalytic and Electrocatalytic

**Figure 13:**
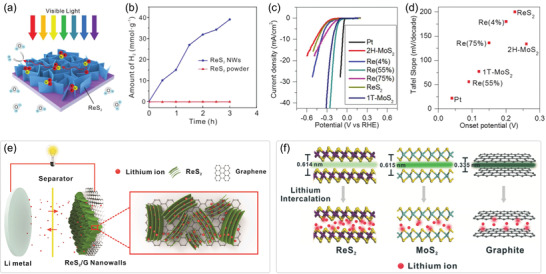
a) Schematic diagram of the two‐electron photocatalytic reaction catalyzed by ReS_2_ nanowalls. b) Photocatalytic hydrogen evolution performance of the ReS_2_ nanowalls and ReS_2_ powder under visible light irradiation (*λ* > 420 nm). Reproduced with permission.^[^ ^26^ ^]^ Copyright 2018, Wiley‐VCH. c) Polarization curves as well as d) tafel slope and onset overpotentials of different Re‐doped MoS_2_ catalysts and reference samples. Reproduced with permission.^[^ ^136^ ^]^ Copyright 2018, Wiley‐VCH. e) Schematic illustration of lithium intercalation in V‐ReS_2_/3DGF‐based lithium‐ion battery. f) Schematic illustration of the weak interlayer interaction of ReS_2_, compared with MoS_2_ and graphite. Reproduced with permission.^[^ ^25^ ^]^ Copyright 2018, Wiley‐VCH.

The suitable bandgap with neglect layer dependence of ReX_2_ enables it to be ideal photocatalyst for high‐efficiency solar‐energy conversion. Based on theory calculations, researchers have found that for monolayer and multilayer ReS_2_, the bandgap and band edge positions are excellently matched with the water‐splitting energy levels. Moreover, the effective masses of the carriers are relatively light, and the optical absorption coefficients are high under visible illumination. Above superior properties suggest that ReS_2_ should be an efficient photocatalyst for water splitting. In experiment, Fu and coworkers demonstrated a two‐electron catalytic reaction to achieve the efficient reduction of two H^+^ (2H^+^ + 2e^−^ → H_2_) utilizing the distinct trion behavior of ReS_2_ (**Figure** **13**,b).^[^ ^26^ ^]^ The free electrons in vertical ReS_2_ can be captured by the tightly bound excitons to form trions. Notably, the extremely weak interlayer interactions of ReS_2_ cause the bulk to behave as electronically decoupled monolayers, which allows ReS_2_ to generate numerous trions, even in a multilayer nanosheet. These trions can migrate to the surface and participate in the two‐electron reaction at the abundant active sites. With this approach, a superior photocatalytic hydrogen evolution rate of 13 mmol g^−1^ h^−1^ was achieved under visible light irradiation, which surpassed the rates of most reported TMDs composite photocatalysts.

Interestingly, the anisotropic structure of ReX_2_ endows them polarization‐dependent photocatalytic degradation behavior.^[^ ^179^ ^]^ The enhanced photocatalytic behavior of the layered ReS_2_ with optical polarization along the *b*‐axis demonstrated that the formation of a nano‐diamond‐chain and a one‐layer trigonal crystalline phase is beneficial for the versatile energy applications of ReX_2_. Moreover, strain engineering of ReX_2_ can effectively turn the band structure and density of states,^[^ ^48^ ^]^ which could be considered as a factor to improve the photocatalytic performance. A theoretical study has predicted that 1–5% y‐axial tensile strain can greatly enhance the performance of single‐layer ReS_2_ for overall photocatalytic water splitting.^[^ ^180^ ^]^ Moreover, a dual‐enhancement in ReSe_2_ nanosheets with high concentration of active sites and efficient use of hot electrons is simultaneously achieved with moderate Mo doping.^[^ ^181^ ^]^ Constructing nanocomposites of ReX_2_ with other semiconductor catalytics to realize the co‐catalytic is another route to elevate its photocatalysts performance.^[^ ^182^, ^183^, ^184^ ^]^ The TiO_2_@ReS_2_ nanocomposites present significantly enhanced degradation activity of organic pigments under sunlight illumination in comparison with pure TiO_2_ nanoparticles.^[^ ^184^ ^]^ The underlying mechanism of enhanced photocatalytic activity was attributed to the improved separation efficiency of photogenerated electron–hole pairs.

Besides the superior photocatalytic performance, ReX_2_ has been considered as an ideal catalyst for the electrocatalytic hydrogen evolution reaction (HER).^[^ ^123^, ^185^ ^]^ On the one hand, the atomic sites on edges of ReS_2_ nanosheets have dangling and unsaturated coordination bonds, which are the primary active sites of HER. On the other hand, the reduced dimension resulting in the extensive exposure of ReX_2_ edges, and thus the specific surface area is enhanced effectively. In particular, as discussed in Section 3.5.3, the unusual out‐of‐plane growth and off‐symmetry growth of ReX_2_ provide more feasibilities to tailored design the materials with diverse structures for high performance HER. For example, Zhang and coworkers synthesized monolayer ReSe_2_ with a sunflower‐like shape on SiO_2_/Si substrate via CVD growth, and then transferred it onto Au foil to construct the ReSe_2_/Au electrode for HER.^[^ ^185^ ^]^ The edge‐abundant sunflower‐shaped ReSe_2_ nanosheets originating from the off‐symmetry growth exhibited superior electrocatalytic HER activity, featuring a relatively low overpotential of 270 mV and Tafel slope of ≈76 mV dec^−1^ and an exchange current density of 10.5 µA cm^−2^, which were superior to those of compact truncated‐triangle‐shaped ReS_2_ nanosheets. Certainly, the catalysis performance of CVD‐grown planar ReX_2_ nanosheet is limited by the tiny amount of electrode materials. Koratkar and coworkers prepared vertically oriented arrays of 3D ReS_2_ nanosheets via CVD growth to increase the amount of electrode materials and the exposed surface area, sharp edges and corners.^[^ ^123^ ^]^ As HER catalysts, the vertically oriented arrays of ReS_2_ provided very small onset overpotential (<100 mV) and an exceptional exchange‐current density (≈67.6 µA cm^−2^), which is vastly superior to the planar ReS_2_ electrode. Besides engineering the edges of ReX_2_ to improve its catalytic activity, how to increase the active site at the basal plane of ReX_2_ is currently a subject of great interest and intensive investigation. DFT calculation has shown that the catalytic activity of the ReS_2_ basal plane can be efficiently activated by doping with transition metal atoms (such as Mo, Cr, Mn, Fe, Co, Pt, Au, and Ag).^[^ ^186^ ^]^ The predicated performance improvement is attributed to the decreased hydrogen adsorption free energy (Δ*G*_H*_) and exposed active sites by introducing more unsaturated electrons. Experimentally, Zhou and coworkers indeed found that the normally inert basal planes of 2D layers of ReS_2_ (MoS_2_) can generate highly catalytic activity for the HER when alloyed with Mo (Re).^[^ ^136^ ^]^ The CVD‐grown 1T′ Re_0.55_Mo_0.45_S_2_ alloy as HER catalyst has a much lower onset potential of 90 mV and a small Tafel slope of 56 mV dec^−1^, which are compared to most of the 1T′ MoS_2_‐based catalysts (Figure 13c,d). Moreover, introducing defect is another effective way to enhance the HER performance of ReS_2_ basal plane.^[^ ^121^, ^122^ ^]^ Abundant Re atoms vacancies were produced by visible‐light assisted pre‐electrolysis treatment, which can effectively decrease the overpotential from 170 to 88 mV compared with defect‐free ReS_2_.^[^ ^122^ ^]^ Theoretical calculations confirmed that the abundant Re atoms vacancies with lower Gibbs free energy for H adsorption activate the inert basal plane as highly active sites for hydrogen evolution.

As is well known, the conductivity of electrode materials is an important parameter for HER performance. From the perspective of improving contact between catalyst and electrode, several works have explored the direct CVD growth of vertically aligned ReX_2_ nanosheets on conductive substrates like carbon cloth and glass carbon to improve the HER performance.^[^ ^123^, ^187^ ^]^ Recently, Pumera and coworkers developed a simple electrodeposition approach using NH_4_ReS_2_ precursor to synthesize the electrocatalyst ReS_2_ on 3D and 2D‐printed carbon electrodes.^[^ ^188^ ^]^ Additionally, the HER performance of ReX_2_ can also be improved by the preparation of composites containing conductive additives, such as ReS_2_@ReO_2_, ReS_2_@TiO_2_, and ReS_2_@RGO (reduced oxidation graphene) composite electrode materials.^[^ ^183^, ^189^, ^190^ ^]^

Overall, these positive results demonstrate that ReX_2_ is a superior dual functional, photo‐ and electrocatalytic material. In the future, further exploring various methods such as heteroatom doping, defect engineering, phase engineering and interface structure optimization is encouraged to advance the catalysis activity of ReX_2_. Moreover, band structure or Fermi energy level tuning should be an effective route to improve the photocatalysis performance. In addition to the preceding applications in photocatalytic and HER, we have faith that ReX_2_ will emerge to extend the scope of applications in other catalytic systems.

#### 4.2.2 Lithium‐Ion Battery

Lithium‐ion battery (LIB), an important energy storage technology, has attracted tremendous attention in recent years owing to its wide applications in portable electronics, power tools and environmentally friendly transportation, and so on.^[^ ^175^ ^]^ Developing alternative anode materials with higher energy density, better rate capability and longer cycling life to replace the traditionally and commercially used graphite is of increasing interest to fulfill the growing demands for LIBs. Among various candidates, 2D layered materials including graphene and TMDs have shown great promise as anode materials of LIBs due to their high conductivity and natural layered structures for ion intercalation.^[^ ^177^ ^]^ In particular, ReX_2_ appears to have great potential in high‐current‐density LIBs because of its extremely weak interlayer coupling compared with graphene and other 2D TMDs materials.^[^ ^37^ ^]^ For instance, the interlayer coupling of ReS_2_ is about 18 meV per unit cell, while that of MoS_2_ is about 460 meV for comparison.^[^ ^13^ ^]^ The weak interlayer coupling and the larger interlayer spacing afford new opportunities for massive alkali‐metal ions to efficiently diffuse without significant volume expansion. This is a highly significant advantage over other 2D layered materials. Theory calculation shows the capacity value for Li^+^ storage of pure ReS_2_ can up to ≈430 mAh g^−1^. As thus, ReX_2_ has been widely utilized as anode material to construct high‐performance LIBs. In this section, we provide an overview of the recent progresses of LIBs based on ReX_2_ electrode materials, including the structure design, composite ingredients, reaction mechanism, and electrochemical performance.

As mentioned in section 3.5.3, vertically oriented 3D ReS_2_ nanosheets can increase catalytic active sites, here, this structural feature can also significantly improve the performance of ReS_2_‐based Li‐S battery.^[^ ^123^ ^]^ The specific capacity of the battery is retained above 750 mAh g^−1^ after 300 cycles with only ≈0.063% capacity decay per cycle, which is much better than the baseline battery (without ReS_2_) with ≈0.184% capacity decay per cycle under the same test conditions. Utilizing the extremely weak van der Waals coupling of ReX_2_, Fu and coworkers demonstrated a high‐current‐density LIBs by synthesizing uniformly distributed vertical ReS_2_ nanowalls (V‐ReS_2_) on 3D graphene foam (3DGF) via CVD growth (Figure 13e,f).^[^ ^25^ ^]^ The 3DGF was used as a template to enhance the conductivity and enlarge the specific surface area of the whole electrode material. This favorable vertical structure shortens the pathways and facilitates the fast diffusion of both Li^+^ and electrolyte ions in the battery system as schematically shown in Figure 13e. As a result, the V‐ReS_2_/3DGF composite exhibited good cycling stability at high‐current‐densities (1000 mA g^−1^) when serving as anode material for LIBs. Especially, at the high current density of 1000 mA g^−1^, its capacity still maintained over 200 mAh g^−1^ even after 500 cycles. To further enhance the conductivity and ion diffusion rate for improving both high‐rate capability and long life, a series of ReS_2_/carbon composite electrode materials were constructed.^[^ ^27^, ^191^, ^192^, ^193^ ^]^ For example, Qiao and coworkers fabricated a rGO@ReS_2_@N‐C composite where ReS_2_ is confined in 2D‐honeycombed carbon nanosheets composed of rGO and N‐doped carbon coating layer.^[^ ^27^ ^]^ The rGO@ReS_2_@N‐C composites achieved rate performance of 231 mAh g^−1^ at 10 A g^−1^, together with ultra‐stable long‐term cycling of 192 mAh g^−1^ at 2 A g^−1^ after 4000 cycles.

Additionally, enlarging the interlayer spacing of ReS_2_ is conducive to the diffusion and migration of alkali‐metal ions through electrode materials. Park and coworkers demonstrated oxygen incorporation and interlayer expansion of ReS_2_ nanosheets deposited on hollow mesoporous carbon spheres, which facilitates the redox kinetics by means of the improved electronic conductivity and rapid ion diffusion.^[^ ^194^ ^]^ With this approach, the composite electrode displayed a stable operation up to 10 A g^−1^ and a resilient recovery of capacity near 318 mAh g^−1^. Besides, the role of crystallographic orientation and anisotropy in the Na^+^ and Li^+^ battery electrochemistry was investigated at the atomic scale via in‐situ TEM.^[^ ^195^ ^]^ It was found that the reaction speed of lithiation/sodiation of ReS_2_ is highly anisotropic, occurring faster along the in‐plane ReS_2_ layer than along the out‐of‐plane direction. This work suggests that crystallographic orientation in the highly anisotropic ReS_2_ electrode materials can be exploited as a design parameter to further improve battery performance.

On the whole, great progresses have been achieved in the applications of ReX_2_ in LIBs, including the synthesis of vertical 3D nanoarrays, the expansion of interlayer spacing, and the enhancement of conductance via constructing various composites, etc. Certainly, there is still considerable room for further elevating the specific capacity and current‐density of ReX_2_‐based LIBs. For instance, developing effective synthesis approach to improve the electrical contact between ReS_2_ and electrode is highly desirable. An in‐depth understanding of ions diffusion and migration along different crystallographic orientations of the anisotropic ReS_2_ is significantly critical to effectively design and synthesize the material with preferential structure architectures. Moreover, the study of battery application of ReSe_2_ is still blank, which is worth exploring in the future.

### 4.3 Other Applications

#### 4.3.1 Raman Enhancement

In the past ten years, 2D materials including graphene and TMDs have been widely used as SERS substrates, which show great application prospect due to their good electrical and thermal conductivity, atomic level flatness, and chemical stability.^[^ ^196^, ^197^, ^198^, ^199^ ^]^ Different from 2H TMDs materials, the 1T′ phase ReX_2_ with low lattice symmetry as a SERS substrate endow more degree of freedom for exploring novel enhancement effects.

**Figure 14:**
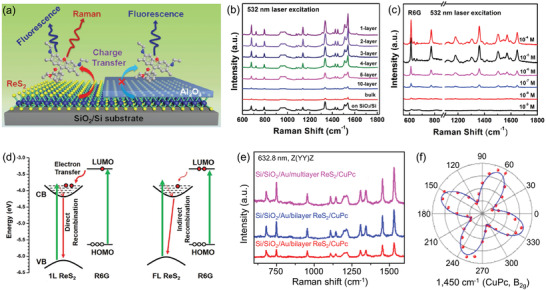
a) Schematic illustration of the experimental setup for understanding the Raman enhancement mechanism on ReS_2_ nanosheets. b) Raman spectra of 1 nm thick CuPc on ReS_2_ films with different layer numbers and SiO_2_/Si substrate. c) Concentration‐dependent Raman spectra of different fluorescent dyes adsorbed on monolayer ReS_2_. Reproduced with permission.^[^ ^200^ ^]^ Copyright 2018, Wiley‐VCH. d) Energy diagram of charge transfer process between R6G molecule and single‐layer (1L) and few‐layer (FL) ReS_2_ nanosheets, reproduced with permission. e) Raman spectra of CuPc molecules on ReS_2_/gold/PMMA/300 nm SiO_2_/Si substrate. f) Polar plots of the normalized intensities of 1450 cm^−1^ (CuPc, B_2g_) modes as a function of sample rotation angle on ReS_2_. Reproduced with permission.^[^ ^202^ ^]^ Copyright 2019, Tsinghua University Press and Springer Nature.

Xu and coworkers adopted a series of probe molecular including phthalocyanine (CuPc), rhodamine 6G, rhodamine B (RhB), crystal violet (CV) and methylene blue (MB) to investigate the Raman enhancement effect and possible enhancement mechanism of ReS_2_ substrate (**Figure** **14**a).^[^ ^200^ ^]^ The Raman signal of CuPc molecular on the ReS_2_ film decreases with the increasing of layer numbers (Figure 14b), indicating strong layer‐number‐dependence of the Raman enhancement effect. The Raman enhancement factor of CuPc on monolayer ReS_2_ is 2.94. Moreover, Raman signals of probe molecule with different concentrations on monolayer ReS_2_ show that the detection limit of R6G and MB molecular as low as 10^−9^ m (Figure 14c), demonstrating the feasibility of utilizing monolayer ReS_2_ as highly sensitive Raman enhancement substrate for detection applications. Furthermore, combining Raman and photoluminescence studies with the assistance of an Al_2_O_3_ dielectric layer strongly suggests that the Raman enhancement effect of molecular on ReS_2_ originates from a charge transfer process rather than from an energy transfer process. Figure 14d presents the energy diagram of charge transfer process between R6G molecule and monolayer as well as few‐layer ReS_2_, in which the degree of charge transfer from R6G to ReS_2_ is heavily impacted by the band structure of ReS_2_. Furthermore, the authors also performed the angle‐resolved polarized Raman spectra of CuPc on monolayer ReS_2_, but they haven't observed obvious polarization‐dependence Raman enhancement effect. This should be attributed to the existence of abundant nanosized subdomains with different lattice orientations in the CVD‐grown ReS_2_ film as demonstrated in the preparation section.

Using mechanically exfoliated high‐quality ReS_2_ as substrate, Zhang and coworkers reported the polarization dependence of the enhanced Raman scattering of CuPc molecules, which is attributed to the different charge transfer probabilities between ReS_2_ and molecules oriented along different crystalline directions.^[^ ^201^ ^]^ To further understand the anisotropic Raman enhancement mechanism of the ReS_2_ substrate, they investigated the doping modulated in‐plane anisotropic Raman enhancement by stacking monolayer ReS_2_ on a flat gold film or graphene, where the electrons doping in ReS_2_ can decrease the anisotropic Raman enhancement.^[^ ^202^ ^]^ The degeneration of anisotropy Raman enhancement was attributed to the electrons transferring from the Fermi level of gold/graphene to the conduction band of ReS_2_, which increased the electrons density in the conduction band and thus decreased the probability of charge transfer from CuPc molecules with major axes parallel to the *b*‐axis of ReS_2_. These unique anisotropic charge interactions between molecules and ReX_2_ not only provide new insights into the chemical enhancement mechanism in SERS, but also suggest new application perspectives in optoelectronics, such as polarization‐controlled molecular electronic switches.

#### 4.3.2 Sensor

2D ReX_2_ materials with high surface‐to‐volume ratio and semiconductor characteristic make them an ideal building block and platform to construct diverse sensors, because changing their surrounding environment can modulate their electronic, optoelectronic and chemical properties.^[^ ^203^ ^]^ Hence, the design and fabrication of ReX_2_‐based materials for sensor applications have intrigued considerable attention, and several novel ReX_2_‐based sensors have been developed recently.^[^ ^28^, ^204^, ^205^ ^]^

**Figure 15:**
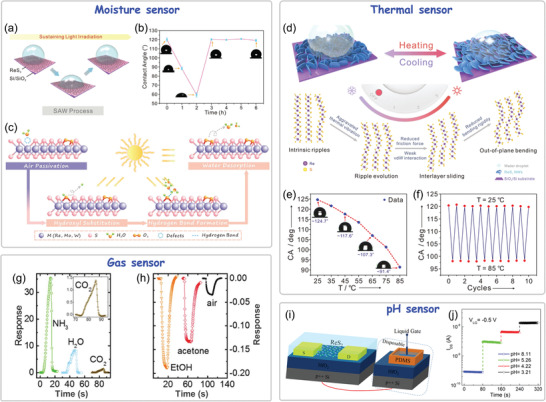
a) Schematic of the SAW process of the ReS_2_ film under sustaining visible light irradiation. b) Contact angle changes of the ReS_2_ film under visible light irradiation. c) Schematic of the SAW process under sustaining visible light irradiation.^[^ ^204^ ^]^ Copyright 2018, Wiley‐VCH. d) Schematic representing the TIB process of the ReS_2_ nanowalls. e) Wetting characteristics of the ReS_2_ nanowalls with increasing temperature. f) The wetting switch is reversible for 10 cycles. Reproduced with permission.^[^ ^28^ ^]^ Copyright 2018, Wiley‐VCH. g,h) Representative gas‐induced time response of the device built with ReS_2_ exposed to different gases. Reproduced with permission.^[^ ^205^ ^]^ Copyright 2019, Wiley‐VCH. i) Schematic diagram of a ReS_2_ FET for pH sensing. j) Response curve of ReS_2_ for pH sensing. Reproduced with permission.^[^ ^206^ ^]^ Copyright 2018, American Chemical Society.

Fu's group presented a smart self‐adapting wettability (SAW) of ReS_2_ under sustaining light irradiation, where the ReS_2_ film was grown on the Si/SiO_2_ substrate via CVD growth.^[^ ^204^ ^]^ **Figure** **15**a shows the schematic diagram of the SAW process, and the corresponding contact angle variation reflects the wetting behavior during the whole process (Figure 15b). The surface of ReS_2_ realized an automatic transition of hydrophobic‐hydrophilic‐hydrophobic state, indicating a self‐adapting wettability behavior. The SAW process could be divided into three considered procedures: hydroxyl substitution, formation of hydrogen bonds, and water desorption (Figure 15c). Notably, the SAW behavior achieved in ReS_2_ breaks the stereotype that a single stimulus leads to a monotonic change in structures or properties. Using the CVD‐grown ReS_2_ nanowalls, Fu and coworkers further presented an interesting temperature‐responsive behavior that was triggered by stable and reversible thermally induced bending (TIB) (Figure 15d).^[^ ^28^ ^]^ During the heating process, deformation and interlayer sliding occur successively in the ReS_2_ nanowalls, which were reflected in the contact angle tests, and its wetting ability increases with increasing temperature (Figure 15e). Notably, the flexibility of ReS_2_ enables its nanosheet to recover to the initial configuration when the temperature drops, and show good cycling stability during multiple TIB process (Figure 15f). Above all, the unique SAW and TIB behaviors provide new insight to broaden the applications of ReX_2_ materials, and open up new perspectives for multifunctional intelligent devices. By combining the solution‐based synthesis with surface functionalization strategies, the feasibility of colloidal ReS_2_ nanosheet films for sensing different gases (such as NH_3_, H_2_O, CO_2_ in Figure 15g, alcohol, acetone and dry air in Figure 15h) is demonstrated with highly competitive performance in comparison with devices built with CVD‐grown ReS_2_ and MoS_2_.^[^ ^205^ ^]^ Moreover, a pH sensor was achieved via a bilayer ReS_2_ FET with HfO_2_ as a sensing oxide (Figure 15i).^[^ ^206^ ^]^ It is observed that I_ds_ increases stepwise with discrete changes in pH value from 8.11 to 3.21 and quickly goes to a stable value at each pH value (Figure 15j), demonstrating the fast response characteristic. The excellent gas and pH sensing performance suggest the great potential of ReX_2_ in hazardous and toxic sensor and biosensor applications. Therefore, ReS_2_ has been demonstrated as a promising material for constructing diverse sensors to respond with temperature, wetness and a few chemicals, which still have considerable room for improvement by exploiting device designs, finely tuned structures and surface modifications.

Besides the above applications, 2D ReX_2_ materials have been demonstrated to show remarkable elastic modulus and large strain before fracture.^[^ ^48^ ^]^ Applying mechanical strain to ReX_2_ can hugely modulate their electronic conduction and optical behavior. Especially, the different crystalline directions of anisotropic ReX_2_ respond differently to the uniaxial strain, which indicates distinct strain‐electronic structure interaction along anisotropic directions. A recent study shows that ReS_2_ has completely opposite (positive and negative) piezoresistance along two principal axes, which differed from any previously reported anisotropic piezoresistive effect in other 2D materials.^[^ ^207^ ^]^ The novel property is ideally useful in accurately sense/recognize multidimensional strain signals for the development of strain sensors, conductance‐switch FETs, electronic skin, human‐machine interfaces, etc. Furthermore, theory calculations show that doping, strain or high pressure of ReX_2_ could induce robust magnetic properties and even superconduction,^[^ ^51^, ^208^, ^209^ ^]^ which provide tremendous potential for novel applications such as ultrasensitive sensors, nanoscale electro‐mechanical systems and spintronic devices for information storage.

## 5 Summary and Perspectives

Within just a few years, there has been a rapid surge of interest and tremendous progress for the attractive ReX_2_ material. Besides the communal properties similar to other 2D TMDs, the unusual 1T′ structure of ReX_2_ endows them abundant exciting physicochemical properties, such as weak interlayer coupling and strong anisotropic properties. As a result, it has been shown to have potential for a wide range of important applications including various solid state devices, SERS, sensors, catalytic hydrogen production and lithium ion batteries. Despite it has achieved considerable progress and been revealed extraordinary application potential, the research about ReX_2_ is still in its infancy stage, and many challenges and opportunities deserve to be further explored. It mainly includes the following aspects:

### 5.1 Properties

The low crystal symmetry of ReX_2_ makes it an anisotropic 2D material which exhibits strong anisotropic electrical, optical, vibrational, thermal, and magnetic properties. These unique properties of ReX_2_ open up excellent potential for designing conceptually new devices where the strong anisotropic properties are required. Certainly, the anisotropy ratio of ReX_2_ needs to be further enhanced to fulfill the real applications, which could be realized by applying strain or coupling with the anisotropic plasma nanostructures. Furthermore, it has been shown that doping metal elements into ReS_2_ is an effective knob for tuning the magnetic properties, which broadens the way to realize new functional devices.

Tuning optical, electrical and magnetic properties of ReX_2_ by strain engineering is still at the theory stage. Strain engineering is a unique technique for tuning physical and chemical properties at nanoscale for 2D materials. For example, wrinkle‐generated local strain in ReS_2_ modulates optical gap, enhances light emission, induces magnetism, and alters the electrical properties. Whilst precise control the intensity, direction and manner (tensile or compression) of applied strain is critical but challenging to the controlled modulate the properties of anisotropic ReX_2_. Obviously, carrying out deep experimental research for ReX_2_ is needed. Furthermore, the weak interlayer coupling of ReX_2_ facilitates the intercalation of functional ions provides a great opportunity to explore the intercalation chemistry, a new research direction to modify the properties of 2D materials.

### 5.2 Preparation

Despite tremendous progress have been achieved in the preparation of ReX_2_, the controlled synthesis of single crystal ReX_2_ still faces a great challenge. As demonstrated in Section 3.5, the Re4‐chain reconstruction easily occurs during the synthesis of ReX_2_, which brings plenty of subdomains and GBs in the CVD‐grown ReX_2_. These GBs seriously degrade the properties of ReX_2_ and thus hinders their application in large‐scale optoelectronic devices. Moreover, the attractive anisotropy feature of ReX_2_ makes their device applications have higher requirements for the lattice orientation than that of other 2D TMDs, as the random orientations of different ReX_2_ domains inevitably bring heterogeneous properties. Hence, developing effective approaches to synthesize large‐scale ReX_2_ single crystal domains with uniform orientation is highly desired, and that should be the primary task in future research work. We believe, once the above goal is achieved, the optical and electrical properties of CVD‐grown ReX_2_ would have huge room to be further improved.

From another point of view, effectively utilizing these GBs would burst out new potential of ReX_2_. As is well known, GBs can provide a platform to explore emerging properties that are not manifest in a single crystal, since atomic displacement at GB can modify its electronic state to a large extent. Many novel properties such as free‐electron creation, intrinsic magnetism, tunable bandgap, excitonic relaxation, memristive effect, and enhanced catalysis have been revealed in the GB of 2H TMDs.^[^ ^210^, ^211^, ^212^, ^213^, ^214^, ^215^ ^]^ As for 1T′ ReX_2_, the abundant GB structures provide rare opportunities to explore more novel properties and tailored design of them with desired properties. In this regard, it's worth further exploring the properties and applications of GBs in ReX_2_ from both theory and experiment aspects.

The unusual and stable 1T′ structure and the distinctive properties of ReX_2_ offer both opportunities and challenges for the integration of them with other TMDs materials to build many novel 2D structures for engineering their phase structures, energy band structures, properties and device applications. Moreover, the naturally out‐of‐plane growth tendency of ReX_2_ provides a great chance to construct many novel 3D nanostructures which are highly desired in catalysis and sensor applications. Though initially progresses have been achieved in all above aspects, they are far away from the expectations, and thus further in‐depth studies are required. All in all, controlled preparation is the key that determines the future of ReX_2_.

### 5.3 Application

Photodetectors based on ReX_2_ show competitive performance compared with other TMDs in terms of the responsivity and EQE, especially in the ability to detect polarized light. Surface modification and plasma treatment were demonstrated to improve device performance. However, this improvement is not sufficient. An in‐depth understanding of the photocurrent and carrier recombination mechanisms in ReX_2_ is needed for further improving the performance of their optoelectronic devices to satisfy the practical applications. Moreover, line dichroism absorption, polarized excitons and selectively tunable optical Stark effects have been experimentally achieved in ReX_2_, which could provide more degree of freedom for designing novel multi‐functional optoelectronic devices. For the energy conversion and storage, there are several possible strategies to further improve their performance: i) increase the active sites via introducing dopant or vacancy, ii) improve the conductivity via constructing heterostructures with other high conductivity materials, iii) optimize the materials structures through effectively utilizing the off‐symmetry growth and out‐of‐plane growth features of ReX_2_ to build 3D vertical arrays with high surface area and large interlayer space. Moreover, ReX_2_ has large exposed surface areas available for the material‐analyte interaction, suitable active sites for effective and selective analyte binding, the ability to transduce the binding events into a detectable signal, as well as good mechanical and processing properties. These features enable ReX_2_ huge potential for diverse sensor applications. As described above, ReS_2_ is currently responsive to moisture and temperature, while other stimuli‐responsive fields such as electricity, gas, as well as living systems‐related taste, sight, touch and hearing urgently need to be explored. Some real‐world smart sensor applications like intelligent wearable electronic devices, biosensors, and printing sensors may need ReX_2_ material to fill this gap. Furthermore, owing to the intrinsic anisotropy in electronic properties, ReX_2_ may realize a dissimilarly synaptic response of multiterminal three‐terminal electrolyte‐gated transistor devices, which exhibits great potential for developing an artificial system to mimic the behaviors of the human brain. Among the above potential applications, the intelligent sensors may take an early lead for practical application, because diverse intelligent sensors are highly required to satisfy the demand of modern life, and they have relatively lower requirements to the key materials compared with that for electronic device like a chip. Certainly, element Re is not earth‐abundant and relatively expensive. In this regard, the techno‐economic feasibility should be considered during developing the applications of Re‐based TMDs. Developing the killer application of ReX_2_ should be encouraged.

In a word, the above features that enable ReX_2_ to be unique and deserve to be studied in depth. Here, ReX_2_ is just a more general representation of other TMDs or those similarly low symmetry 2D materials. We believe that this review article will promote the exploration of similar 2D materials and accelerate the development of nanomaterials science.

## Conflict of Interest

The authors declare no conflict of interest.
